# *Fusobacterium nucleatum*-triggered neutrophil extracellular traps facilitate colorectal carcinoma progression

**DOI:** 10.1186/s13046-023-02817-8

**Published:** 2023-09-09

**Authors:** Xuehua Kong, Yu Zhang, Linwei Xiang, Yan You, Yaqian Duan, Yuqing Zhao, Shue Li, Rui Wu, Jiangbo Zhang, Lan Zhou, Liang Duan

**Affiliations:** 1 Department of Laboratory Medicine, Key Laboratory of Laboratory Medical Diagnostics, Ministry of Education, Chongqing Medical University, No. 1 of Yixueyuan Road, Yuzhong District, Chongqing, 400016 China; 2 Department of Laboratory Medicine, The Second Affiliated Hospital of Chongqing Medical University, No.74 Linjiang Road, Yu Zhong District, Chongqing, 400010 China; 3 Department of Pathology, The Second Affiliated Hospital of Chongqing Medical University, Chongqing, 400010 China; 4 Department of Academic Research, The Second Affiliated Hospital of Chongqing Medical University, Chongqing, 400010 China; 5 Department of Laboratory Medicine, The First Affiliated Hospital of Chonqing Medical University, Chongqing, 400016 China; 6 Department of Gastrointestinal Surgery, The Second Affiliated Hospital of Chongqing Medical University, Chongqing, China

**Keywords:** *Fusobacterium nucleatum*, Colorectal carcinoma, Neutrophil extracellular traps, ROS, TLR4, NOD1/2

## Abstract

**Background:**

*Fusobacterium nucleatum* (*Fn*) acts as a procarcinogenic bacterium in colorectal carcinoma (CRC) by regulating the inflammatory tumor microenvironment (TME). Neutrophil extracellular traps (NETs), which can be generated by persistent inflammation, have been recently considered to be significant contributors in promoting cancer progression. However, whether NETs are implicated in *Fn*-related carcinogenesis is still poorly characterized. Here, we explored the role of NETs in *Fn*-related CRC as well as their potential clinical significance.

**Methods:**

*Fn* was measured in tissue specimens and feces samples from CRC patients. The expression of NET markers were also detected in tissue specimens, freshly isolated neutrophils and blood serum from CRC patients, and the correlation of circulating NETs levels with *Fn* was evaluated. Cell-based experiments were conducted to investigate the mechanism by which *Fn* modulates NETs formation. In addition, we clarified the functional mechanism of *Fn*-induced NETs on the growth and metastasis of CRC in vitro and in vivo experiments.

**Results:**

Tissue and blood samples from CRC patients, particularly those from *Fn*-infected CRC patients, exhibited greater neutrophil infiltration and higher NETs levels. *Fn* infection induced abundant NETs production in in vitro studies. Subsequently, we demonstrated that *Fn*-induced NETs indirectly accelerated malignant tumor growth through angiopoiesis, and facilitated tumor metastasis, as manifested by epithelial-mesenchymal transition (EMT)-related cell migration, matrix metalloproteinase (MMP)-mediated basement membrane protein degradation, and trapping of CRC cells. Mechanistically, the Toll-like receptor (TLR4)-reactive oxygen species (ROS) signaling pathway and NOD-like receptor (NOD1/2)-dependent signaling were responsible for *Fn*-stimulated NETs formation. More importantly, circulating NETs combined with carcinoembryonic antigen (CEA) could predict CRC occurrence and metastasis, with areas under the ROC curves (AUCs) of 0.92 and 0.85, respectively.

**Conclusions:**

Our findings indicated that *Fn*-induced NETs abundance by activating TLR4-ROS and NOD1/2 signalings in neutrophils facilitated CRC progression. The combination of circulating NETs and CEA was identified as a novel screening strategy for predicting CRC occurrence and metastasis.

**Supplementary Information:**

The online version contains supplementary material available at 10.1186/s13046-023-02817-8.

## Introduction

Colorectal carcinoma (CRC) accounts for approximately 10% of commonly diagnosed cancers and annual cancer-related deaths worldwide, ranking third in incidence among all malignant tumors worldwide and second in common cancer-related mortality [1, 2]. The pathogenesis of CRC involves various factors such as genetics, lifestyle, obesity and the gut microbiota, among which, the gut microbiota plays an important role during the initiation and progression of CRC [3, 4]. Alterations in the typical gut microbiome have been found to facilitate various processes including metabolic alterations, immune cell activation and proinflammatory responses, which interact to establish a microenvironment conducive to tumor development [5, 6]. However, the mechanisms by which the key CRC-related oncogenic bacteria facilitate CRC progression as well as the underlying clinical significance of the phenomenon still need to be fully studied.

Emerging lines of evidence have suggested that *Fusobacterium nucleatum* (*Fn*) is a major tumor-promoting bacterium, is highly enriched in CRC and associated with the proliferation, immune escape, recurrence, and chemoresistance of CRC [7, 8]. *Fn* infection results in impaired antitumor immunity through the regulation of multiple types of immunocytes, including T cells, NK cells and macrophages, in the TME [9, 10]. We previously observed the tumor-promoting effect of *Fn* mediated through the regulation of S100A9-induecd M2-like macrophage polarization and myeloid-derived suppressor cells (MDSCs) activation [11, 12]. Recently, one mouse study of CRC revealed that *Fn* enrichment was linked with tumor-associated neutrophil (TAN) infiltration [13]. In addition, TGF-β signaling, the key signaling pathway regulating TAN differentiation, was reported to be activated by *Fn* infection [14]. However, the detailed effect of *Fn* on neutrophils in CRC needs to be further studied.

Infection-triggered inflammation constitutes a driving force for neoplastic initiation and processes, enabling infiltrating and resident mutitype inflammatory cells to exert protumorigenic effects. Neutrophil infiltration was independently associated with poor prognosis in the majority of tumours, including CRC [15]. Neutrophil extracellular traps (NETs), net-like structures composed of DNA-histone complexes and proteins released by activated neutrophils, were initially found to play a fundamental role in antimicrobial defense and immune regulation [16–18]. Currently, there are extensive studies showing elevated levels of NETs in tumors and their stimulatory effect on cancer cell malignancy [18, 19]. However, the underlying mechanisms of neutrophil activation and NETs formation in CRC have rarely been researched. Given that NETs released by neutrophils is mainly induced by inflammatory stimuli and extracellular microorganisms, it is not clear whether *Fn* has the capacity to regulate the formation of NETs to establish a cancer-promoting microenvironment in order to potentiate CRC progression.

In the present study, we determined NETs levels in a cohort of CRC patients and analyzed their potential clinical significance. In addition, the function and molecular mechanisms of *Fn* in NETs formation and the contributory roles of NETs in CRC growth and metastasis in cell and mouse models were investigated. In the present study, we demonstrated that *Fn* regulated the formation of NETs and thereby facilitated CRC malignant progression by regulating angiopoiesis, growth and metastasis. The present findings also highlight the clinical significance of circulating NETs, especially combined with CEA, as a screening strategy for predicting CRC occurrence and metastasis.

## Materials and methods

### Patients and clinical samples

The peripheral blood and feces samples from a total of 95 CRC patients, 29 colorectal polyps (CRP) patients and 56 healthy controls (HCs) were enrolled in this study between February 2022 and January 2023 at the Second Affiliated Hospital of Chongqing Medical University. We divide these 95 CRC patients into high *Fn* (*Fn*^−high^) and low *Fn* (*Fn*^−low^) groups based on the median of *Fn* quantification in CRC feces samples. Additionally, CRC tissue samples and matching adjacent normal tissues were also obtained from 34 CRC patients who had undergone surgical resection. These 34 CRC patients were also divided into high *Fn* (*Fn*^−high^) and low *Fn* (*Fn*^−low^) patients according to the number of *Fn* in CRC tissues. Eight normal colorectal tissues were collected from healthy controls who underwent colorectal biopsy to exclude malignancy. Written informed consent was obtained from all participants, and the protocol was approved by the Ethical Committee of the Second Hospital affiliated with Chongqing Medical University (No.2022121). Patient characteristics are shown in Table 1.

**Table 1:** The clinical characteristics of enrolled individuals in this study

Characteristic	Serum specimen			Tissue specimen	
	CRC (*n* = 95)	CRP (*n* = 29)	HC (*n* = 56)	CRC (*n* = 34)	HC (*n* = 8)
**Gender**					
Male (n,%)	52 (54.7%)	17 (58.6%)	24 (42.8%)	21 (61.8%)	6 (75%)
Female (n,%)	43 (45.3%)	12 (41.4%)	32 (57.2%)	13 (38.2%)	2 (25%)
**Age**					
< 60 (n,%)	38 (40%)	14 (48.3%)	50 (89.3%)	16 (47.1%)	3 (37.5%)
≥ 60 (n,%)	57 (60%)	15 (51.7%)	6 (10.7%)	18 (52.9%)	5 (62.5%)
**Location**					
Colon (n,%)	47 (49.5%)	24 (80%)	NA	12 (35.3%)	NA
Rectum (n,%)	48 (50.5%)	5 (20%)	NA	22 (64.7%)	NA
**Differentiation**					
Poorly (n,%)	31 (32.6%)	NA	NA	8(23.5%)	NA
Well + Moderately (n,%)	64 (67.4%)	NA	NA	26(76.5%)	NA
**TNM stage**					
I + II (n,%)	45 (47.4%)	NA	NA	11 (32.3%)	NA
III + IV (n,%)	50 (52.6%)	NA	NA	23 (67.6%)	NA
**Metastasis**					
Present (n,%)	50 (52.6%)	NA	NA	14 (41.2%)	NA
Absent (n,%)	45 (47.4%)	NA	NA	20 (58.8%)	NA

*Abbreviations*: *N/A* not applicable, *n* number of samples, *CRC* colorectal carcinoma, *CRP* colorectal polyps

### Cells culture and bacterial strains culture

Human CRC cell lines HCT116 and SW480, human umbilical vein endothelial cell line HUVEC were conserved at Key Laboratory of Laboratory Medical Diagnostics, Ministry of Education, Chongqing Medical University (Chongqing, China). All the cell lines maintained in Dulbecco’s modified Eagle’s medium (DMEM, Gibco, Grand Island, New York, USA) containing 10% fetal bovine serum (FBS, HyClone, Logan, Utah, USA), 1% penicillin/streptomycin (P/S, HyClone, Logan, Utah, USA) at 37 °C in a 5% CO2, 95% air incubator. *Fusobacterium nucleatum* strain ATCC 25586 were cultured in brain heart infusion (BHI) with hemin, K2HPO4, Vitamin K1, and L-Cysteine under anaerobic conditions at 37 °C as described before [11]. Bacteria were collected by centrifugation at 2500 rpm for 10 min and washed twice with sterile PBS, then adjust its concentration and brought to an OD600 of 1 (∼10^9^ CFU ml^−1^).

### Reagents and antibodies

The primary antibodies used for this study were as follows: anti-Ki67 antibody (ab16667, Abcam, Cambridge, England, UK), anti-CD31 antibody (ab182981, Abcam, Cambridge, England, UK), anti-VEGF antibody (CY5096, Abways, Minhang District, Shanghai, China), anti-MMP9 antibody (ab283575, Abcam, Cambridge, England, UK), anti-MMP2 antibody (ab92536, Abcam, Cambridge, England, UK), anti-CD66b antibody (ab197678, Abcam, Cambridge, England, UK), anti-MPO antibody (66177-Ig, Proteintech, Wuhan, Hubei, China), anti-NE antibody (89241, CST, Boston, Massachusetts, USA), anti-E-cadherin antibody (3195S, SCT, Boston, Massachusetts, USA), anti-vimentin antibody (sc-6260, Santa Cruz, Dallas, Texas, USA), anti-citrullinated modification of histone 3 (CitH3) antibody (ab5103, Abcam, Cambridge, England, UK), anti-peptidyl arginine deiminase 4 (PAD4) antibody (GTX113945, geentex, San Antonio, Texas, USA), anti-N-cadherin antibody (YM0465, Immunoway, Plano, Texas, USA), anti-β-actin (Zoonbio Biotechnology, Nanjing, Jiangsu, China). The inhibitors used for this study were as follows: TLR4 inhibitor TAK-242 (10 μmol/L, S7455, Selleck, Houston, Texas, USA), ROS inhibitor DPI (10 μmol/L, S8639, Selleck, Houston, Texas, USA), NOD1 inhibitor ML130 (20 ug/mL, HY-18639, MedChemExpress, New Jersey, USA), NOD2 inhibitor GSK717 (20 ug/mL, HY136555, MedChemExpress, New Jersey, USA).

### DNA extraction and *Fn* quantification

Bacteria DNA were extracted from clinical fecal samples with QIAamp DNA Mini Kit (51,304, QIAGEN, Hilden, Germany). The amplification and detection of *Fn* was conducted by quantitative real-time PCR. Relative abundance was calculated by -ΔCt method. Total bacterial gene was used as internal reference gene. The primer sets used were:

- *Fn* (Forward): 5'-CAACCATTACTTTAACTCTACCATGTTCA-3';
- *Fn* (Reverse): 5'-GTTGACTTTACAGAAGGAGATTATGTAAAAATC-3';
- Total bacterial (Forward): 5'-GCAGGCCTAACACATGCAAGTC-3';
- Total bacterial (Reverse): 5'-CTGCTGCCTCCCGTAGGAGT-3'.

The median is a cut-off point, which divides patients into *Fn*^−low^ groups and *Fn*^−high^ groups.

### Human Neutrophils Isolation and NETs Formation Assay

Neutrophils were isolated from healthy volunteers and CRC patients by human peripheral blood neutrophils separator kit (P9040, Solarbio, Tongzhou, Beijing, China) according to the manufacturer’s instruction. Briefly, neutrophils separating medium were added to a 15 mL tube. Then, 4 ml fresh anticoagulant whole blood were layered over the separating medium carefully and centrifuged at 1000 × g for 40 min at room temperature. The lower neutrophils band was collected to another fresh 15 ml tube. After erythrocyte lysis and a series of PBS washing, isolated neutrophils were resuspended in RPMI 1640 without FBS.

NETs formation assay to purify NETs was performed as previously described [20]. Freshly isolated neutrophils were stimulated with Phorbol 12-myristate 13-acetate (PMA, 50 ng/ml, Sigma, Saint Louis, Missouri, USA) and incubated for 4 h at 37 °C. Neutrophils and released NETs at the bottom were collected by 4 °C centrifugation at 450 × g for 10 min. Then, NETs-rich supernatant was obtained, followed by centrifuging at 18,000 × g at 4 °C for 10 min. NETs suspension was prepared by cold PBS after supernatant was discarded. Finally, NETs-DNA concentration was measured usinga NanoDrop spectrophotometer (ThermoFisher). Purified NETs in the following experiments for treatment of the cells were used at 500 ng/mL.

### Conditioned medium

Isolated neutrophils were seeded in 24-well plates and then stimulated with *Fn* at multiplicity of infection (MOI) of 100:1, PMA (50 ng/ml, Sigma, Saint Louis, Missouri, USA)) or *DNase I* (100 U/mL, SLBV9316, Sigma, Saint Louis, Missouri, USA) for 4 h at 37 °C and the supernatant was harvested. After centrifugation to remove the bacteria, cells and cellular debris, various conditioned medium (CM) was collected and stored at -80 °C refrigerator for further use.

### Cell migration and invasion assay

In vitro migration and invasion assays were examined in 8 μm transwell cell co-culture chambers coated with or without Matrigel (356,234; Corning; Corning, New York, USA). 1 × 10^5^ CRC cells for migration and 2 × 10^5^ CRC cells for invasion were seeded into the upper chamber in serum-free DMEM, and 5 × 10^5^ neutrophils with or without *DNase I* were seeded in the lower chamber, *Fn* or PMA were used as stimulus as indicated. After 48 h incubation at 37 °C, the non-invading tumor cells were wiped off, and the cells on the bottom side of the membrane were fixed in methanol and stained with crystal violet. The stained cells were quantified in 5 random fields under an inverted microscope at amagnification of × 100.

### Immunohistochemistry (IHC)

Briefly, paraffin-embedded tissue sections were deparaffinized, dehydrated, heated in a pressure pot for 10 min to retrieve antigens, and then treated with 0.3% H_2_O_2_. Thereafter, The sections were incubated with anti-Ki67 (1:200, ab16667, Abcam, Cambridge, England, UK), anti-CD31 (1:2000, ab182981, Abcam, Cambridge, England, UK), anti-VEGF (1:200, CY5096, Abways, Minhang District, Shanghai, China), anti-MMP9 (1:5000, ab283575, Abcam, Cambridge, England, UK), anti-MMP2 (1:200, ab92536, Abcam, Cambridge, England, UK), anti-CD66b (1:200, ab197678, Abcam, Cambridge, England, UK), and followed with secondary antibody incubation tagged with the peroxidase enzyme (1:200, SP-9001, Zhongshan Golden Bridge, Haidian, Beijing, China) for 30 min. The slides were finally visualized with 0.05% DAB and were counterstained with hematoxylin and then observed using Nikon E400 Light Microscope.

### Immunofluorescence (IF)

For cell IF staining, 5 × 10^5^ pretreated neutrophils or 4 × 10^4^ CRC cells were seeded on climbing piece in 24-well plates for 4 h. Cells were fixed with 4% paraformaldehyde for 15 min, washed twice with PBS, and permeabilized with 0.1% Triton X-100 for 10 min. Cells were then blocked with 10% goat serum for 1 h and incubated with anti-MPO antibody (1:200, 66,177-Ig, Proteintech, Wuhan, Hubei, China), anti-NE antibody (1:400, 89,241, CST, Boston, Massachusetts, USA), anti-E-cadherin (1:200, 3195S, SCT, Boston, Massachusetts, USA) and anti-vimentin antibodies (1:500, sc-6260, Santa Cruz, Dallas, Texas, USA) at 4 °C overnight. The next day, cells were rinsed with PBS and then incubated with Alexa Fluor 594-conjugated goat anti-mouse secondary antibody (1:200, A23410, Abbkine, Wuhan, Hubei, China) or Alexa Fluor 488-conjugated goat anti-rabbit secondary antibody (1:200, A23220-10001, Abbkine, Wuhan, Hubei, China) for 1 h in the dark, and then washed with PBS, and counterstained the nucleus with Hoechst 33,258 (1:100, C0021, Solarbio, Tongzhou, Beijing, China) for 10 min. After washing with PBS, slides were mounted with antifade polyvinylpyrrolidone mounting medium (Beyotime, Songjiang, Shanghai, China), Images were observed with confocal microscope (Leica, Germany).

For tissue IF staining, paraffin-embedded sections were proceeded with deparaffinization, rehydration, and antigen retrieval. The sections were incubated with anti-MPO (1:200, Proteintech, Wuhan, Hubei, China), anti-CitH3 (1:100, Abcam, Cambridge, England, UK) antibodies, followed by Alexa fluor 594-conjugated anti-mouse IgG and Alexa fluor 488-conjugated anti-rabbit IgG secondary antibodies. Tissue sections were also stained with DAPI and visualized under a multi-laser confocal microscope (Leica, Germany).

### Western blot

Cells or tissues with different treatments were collected and lysed with RIPA buffer containing phosphatase/protease inhibitor to obtain total cellular protein. Equal amounts of proteins (30 µg) were subjected to SDS-PAGE and transferred to PVDF membranes. The membranes were blocked with 5% BSA for 2 h and incubated with primary antibodies overnight at 4 °C. Primary antibodies including anti-CitH3 (1:1000, ab5103, Abcam, Cambridge, England, UK), anti-PAD4 (1:1000, GTX113945, geentex, San Antonio, Texas, USA), anti-N-cadherin (1:1000, YM0465, Immunoway, Plano, Texas, USA), anti-E-cadherin (1:1000, 3195S, CST, Boston, Massachusetts, USA), anti-vimentin (1:1000, sc-6260, Santa Cruz, Dallas, Texas, USA), anti-VEGF (1:200, CY5096, Abways, Minhang District, Shanghai, China), anti-MMP9 (1:1000, ab283575, Abcam, Cambridge, England, UK), anti-MMP2 (1:1000, ab92536, Abcam, Cambridge, England, UK), and anti-β-actin (1:1000, Zoonbio Biotechnology, Nanjing, Jiangsu, China), followed by incubation with horseradish-peroxidase-conjugated secondary antibodies for 1 h at 37 °C. The peroxidase activity of secondary antibodies was detected with ECL Western blot substrate and recorded by Bio-Rad Electrophoresis Documentation (ECL, Millipore, Germany).

### RNA extraction and Quantitative Real-Time PCR

Total RNA from the treated cells was obtained with TRIzol reagents (Invitrogen, Carlsbad, California, USA). cDNA was reversed by with a Reverse Transcription kit (Takara, Japan). The mRNA levels of NOD1, NOD2 and TLR4 were analyzed and normalized to the GAPDH with the CFX96 real-time PCR detection system (Bio-Rad, USA) using SYBR Green dye (Biomake, Houston, Texas, USA). The following primer pairs were used:

- NOD1 (Forward): 5'-CAGGTCTCCGAGAGGGTACTG-3';
- NOD1 (Reverse): 5'-TGTGTCCATATAGGTCTCCTCCA-3';
- NOD2 (Forward): 5'-ACCTTTGATGGCTTTGACG-3';
- NOD2 (Reverse): 5'-CACCTTGCGGGCATTCTT-3';
- TLR4 (Forward): 5'-AGAATGCTAAGGTTGCCGCT-3';
- TLR4 (Reverse): 5'-CTATCACCGTCTGACCGAGC-3';
- GAPDH (Forward): 5'-ACAACTTTGGTATCGTGGAAGG-3';
- GAPDH (Reverse): 5'-GCCATCACGCCACAGTTTC-3'.

### Cell adhesion assay

Neutrophils (1 × 10^6^ cells) were plated in a 24-well plate and stimulated with PMA (50 ng/ml) for 4 h. HCT116 and SW480 cells (5 × 10^5^ cells) were stained with Dil (Beyotime, China). Then Dil-labeled cells were added to the wells and cultured for 20 min at 37 °C in a 5% CO_2_ atmosphere. After two times PBS washing and 4% paraformaldehyde fixation, the nucleus was stained with Hoechst 33,258 (1:100, C0021, Solarbio, Tongzhou, Beijing, China) for 10 min. The attached cells were visualized under a light microscope at a magnification of × 200.

### Tube formation assay

50 μL Matrigel (356,234; Corning; Corning, New York, USA) was planted into 96-well plates and incubated for 1 h at 37 °C. Then HUVEC cells were suspended in corresponding conditioned medium and 2.5 × 10^4^ cells were inoculated in each well. 4 h later, tube formation was observed under light microscope and analyzed by Image J.

### Cell proliferation assay

Cell proliferation was assessed using a Cell Counting Kit. 2 × 10^3^ HCT116 and SW480 cells were seeded on 96-well plates and incubated for 6 h, then cultured with corresponding conditioned medium. 10 uL CCK8 solution was added. The absorbance was then measured daily using a microplate reader at 450 nm.

### Fluorescence In Situ Hybridization (FISH) analysis

*Fn* in human CRC tissue and mouse CRC tissue was detected using *Fn*-targeted probe, FUS664 (FITC-labeled), 5′-CTT GTA GTT CCG C(C/T) TAC CTC-3′ [21]. The FISH method was performed according to the protocol previously described. Five random fields per slide were observerd at a magnification of × 200, then, the average number of bacteria each field was calculated. The average number of *Fn* in each field in the CRC tissue of < 20 and > 20 visualized FUS664 probes was defined as *Fn*^−low^ and *Fn*^−high^ abundance, respectively.

### ELISA

MPO-DNA complexes were evaluated using a capture ELISA with the commercial cell death detection ELISA kit (11,774,425,001, Roche, Mannheim, Germany). 5 ug/ml anti-MPO antibody (0400–0002, ABD Serotec, Hercules, California, USA) was coated to 96-well microtiter plates overnight at 4 °C. The next day each well was blocked in 1% BSA, then 40 µL serum was added and incubated at RT on a shaking device (320 rpm) for 2 h according to the manufacturer´s instructions. After washing three times with 250 µL incubation buffer per well, 100 ul peroxidase substrate (ABTS) was added and incubated on a shaking device (200 rpm) in the dark for 40 min. The absorbance at 405 nm was measured using a microplate reader.

### Electrochemiluminescence immunoassay

The CEA values in the blood samples were measured by electrochemiluminescence immunoassay “ECLIA” using (Roche cobas e411, Switzerland) with reference to previously described methods [22].

### Flow Cytometry (FCM)

The production of ROS in neutrophils of different treatment groups were analyzed using the dichlorodihydrofluorescein diacetate (H2DCFDA) probe (S9687, Selleck, Houston, Texas, USA) by flow cytometry. Neutrophils (Neu) were stimulated with *Fn*, PMA, *Fn* + TAK-242, *Fn* + ML130, or *Fn* + GSK717 for 6 h, then cells were collected and washed with PBS and then stained with 10 µM H2DCFDA for 30 min at 37 °C. Finally, after washing off residual H2DCFDA, cells were analyzed using flow cytometry (CytoFLEX). To investigate apoptosis of CRC cells, which had been treated with (Neu)-CM, (Neu + *Fn*)-CM, (Neu + *Fn* + *DNase I*)-CM, (Neu + PMA)-CM or NETs for 48 h, Dead Cell Apoptosis Kit (Life Technologies) with Annexin V / FITC and PI was used according to the manufacturer’s recommendation. All the experiments were performed three times.

### AOM/DSS Model

Seven-week-old male C57BL/6 mice were purchased from Laboratory Animal Research in Chongqing Medical University. Mice were randomly divided into three groups (*n* = 5 in each group) and housed under controlled SPF conditions. After 7 days of acclimatization, the mice were intraperitoneally injected with AOM (A5486, Sigma-Aldrich, Saint Louis, Missouri, USA) at a dose of 12 mg/kg. Seven days later, the mice were given 2.5% DSS (216,011,080, MP Biomedicals, Santa Ana, California, USA) dissolved in drinking water for 7 days, followed by 14 days of regular drinking water for recovery. The same cycle was repeated three times. In every cycle, *Fn* (1 × 10^9^ CFU) was administered by gavage two times during the repeated water recovery, and *DNase I* were intraperitoneally injected every three days until the mice were sacrificed. In addition, some other C57BL/6 mice were randomly divided into two groups (*n* = 4 in each group). Based on the same AOM and DSS treatment as above mentioned, mice were treated with or without purified NETs by intraperitoneal injection every three days. The mice were sacrificed on 12 weeks, colon tissues were separated, and the lengths of the tissues were measured. The colons were dissected on the main longitudinal axis and washed with PBS. The number of tumors in colons was counted and recorded. Part of the tissues were fixed with 4% paraformaldehyde for histopathological examination and immunohistochemical analysis. All the animal procedures were approved by the Animal Ethics Committee of Chongqing Medical University (No.2022126).

### In vivo Metastasis assay

Six-week-old BALB/c nude mice obtained from Gembio (Chengdu, Sichuan, China) randomly were divided into three groups (*n* = 4 in each group). HCT116 cells (1 × 10^6^ cells / each mouse) treated with *Fn* (1 × 10^9^ CFU) for 48 h and then were injected into the tail vein of mice, then DNase I (100 U/mL, SLBV9316, Sigma, USA) was administered by tail vein injection every other day. In addition, some other BALB/c nude mice were randomly divided into two groups (*n* = 5 in each group). HCT116 cells (1 × 10^6^ cells / each mouse) were treated with and without purified NETs for 48 h, and suspended in 100 µL PBS and then injected into their tail vein. The mice were sacrificed after 30 days, and their serum and lungs were collected for ELISA analysis and histopathological examination analysis, respectively. All the animal procedures were approved by the Animal Ethics Committee of Chongqing Medical University (No.2022126).

### Statistical analysis

Statistical analysis was done using Graphpad statistical software (version 5.0). Statistical analysis of MPO-DNA levels in the serum of patients was determined by the Kruskal–Wallis or Mann–Whitney test. The statistical differences between groups were evaluated by Student’s t-test for two groups and oneway ANOVA followed by Newman-Keuls’ multiple comparison test for three or more groups. A *p* value < 0.05 was considered significant.

## Results

### NETs formation is enhanced in CRC, especially in ***Fn***^−high^ CRC

**Fig. 1:**
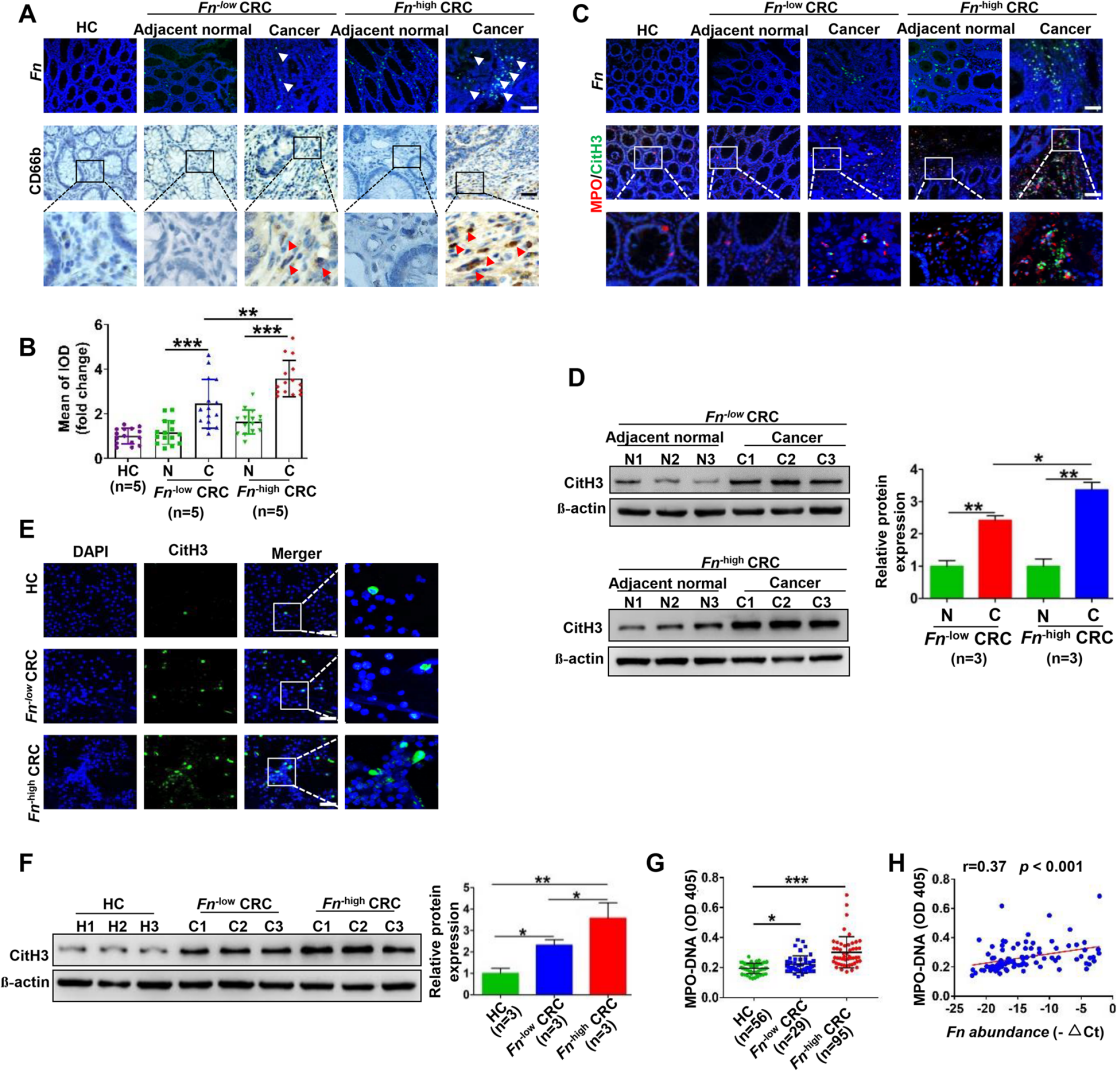
NETs formation is enhanced in CRC, especially in *Fn*^−high^ CRC. **A** Representative images of FISH detection for *Fn* and representative IHC staining for CD66b in tissue sections from HCs, *Fn*^−low^ and *Fn*^−high^ CRC patients and their matched adjacent normal tissues. **B** The mean of IOD for CD66b IHC staining was analyzed utilizing the Image Pro Plus software. Data were quantified by densitometry calculated from 3 random fields of each tissue section from randomly selected HCs (*n* = 5), *Fn*^−low^ (*n* = 5) CRC patients, *Fn*^−high^ (*n* = 5) CRC patients and their matched adjacent normal tissues and are shown as fold changes relative to the HCs. **C** Representative images of FISH detection for *Fn* and representative IF images for MPO (red) and CitH3 (green) in tissue sections from HCs, *Fn*^−low^ and *Fn*^−high^ CRC patients and their matched adjacent normal tissues. **D** Western blot analysis of CitH3 expression in tissue samples from *Fn*^−low^ (*n* = 3) and *Fn*^−high^ (*n* = 3) CRC patients and their matched adjacent normal tissues. CitH3 protein levels were quantified by using densitometry and normalized to β-actin and are shown as fold changes compared to the control (Right). **E** Representative IF images for CitH3 in freshly isolated neutrophils from HCs, *Fn*^−low^ and *Fn*^−high^ CRC patients. **F** Western blot analysis of CitH3 expression in freshly isolated neutrophils from HCs (*n* = 3), *Fn*^−low^ (*n* = 3) and *Fn*^−high^ (*n* = 3) CRC patients. CitH3 protein levels were quantified by using densitometry and normalized to β-actin and are shown as fold changes compared to the control (Right). **G** ELISA analysis for MPO-DNA in the serum from HCs (*n* = 56), *Fn*^−low^ (*n* = 48) and *Fn*^−high^ (*n* = 47) CRC patients. **H** Correlation between serum MPO-DNA levels and -ΔCt of *Fn* quantification in the clinical fecal samples from CRC patients. White arrow: *Fn;* red arrow: CD66b.^+^ neutrophil. White scale bars: 100 μm; Black scale bars: 50 μm. *p < 0.05, **p < 0.01, ***p < 0.001

FISH and IHC were performed to evaluate the enrichment of *Fn* and intrahepatic CD66b^+^ neutrophil infiltration in tissues from HCs and patients with *Fn*^−low^ and *Fn*^−high^ CRC, respectively. There was more CD66b^+^ neutrophil infiltration in CRC tissues than in the corresponding adjacent tissues in patients with either *Fn*^−low^ or *Fn*^−high^ CRC, while *Fn*^−high^ CRC exhibited more CD66b^+^ neutrophil infiltration than *Fn*^−low^ CRC (Fig. 1A and B). DNA/MPO/CitH3, specific markers for NET formation, were analyzed by IF staining to detect and visualize NET formation in these tissues. Similarly, NET levels were elevated in CRC tissues, while *Fn*^−high^ CRC tissues contained more NETs than *Fn*^−low^ CRC tissues (Fig. 1C). Consistent with the NET status in tissue sections, we observed increased protein levels of CitH3 in freshly isolated histiocyte lysates (Fig. 1D) and in freshly isolated neutrophils (Fig. 1E and F). Moreover, the serum levels of MPO-DNA, a marker of circulating NETs, were also increased especially in patients with *Fn*^−high^ CRC (Fig. 1G). Most notably, we observed a close correlation between serum MPO-DNA levels and *Fn* DNA levels in feces in these CRC patients (Fig. 1H).

### *Fn* stimulates NETs formation in vitro

**Fig. 2:**
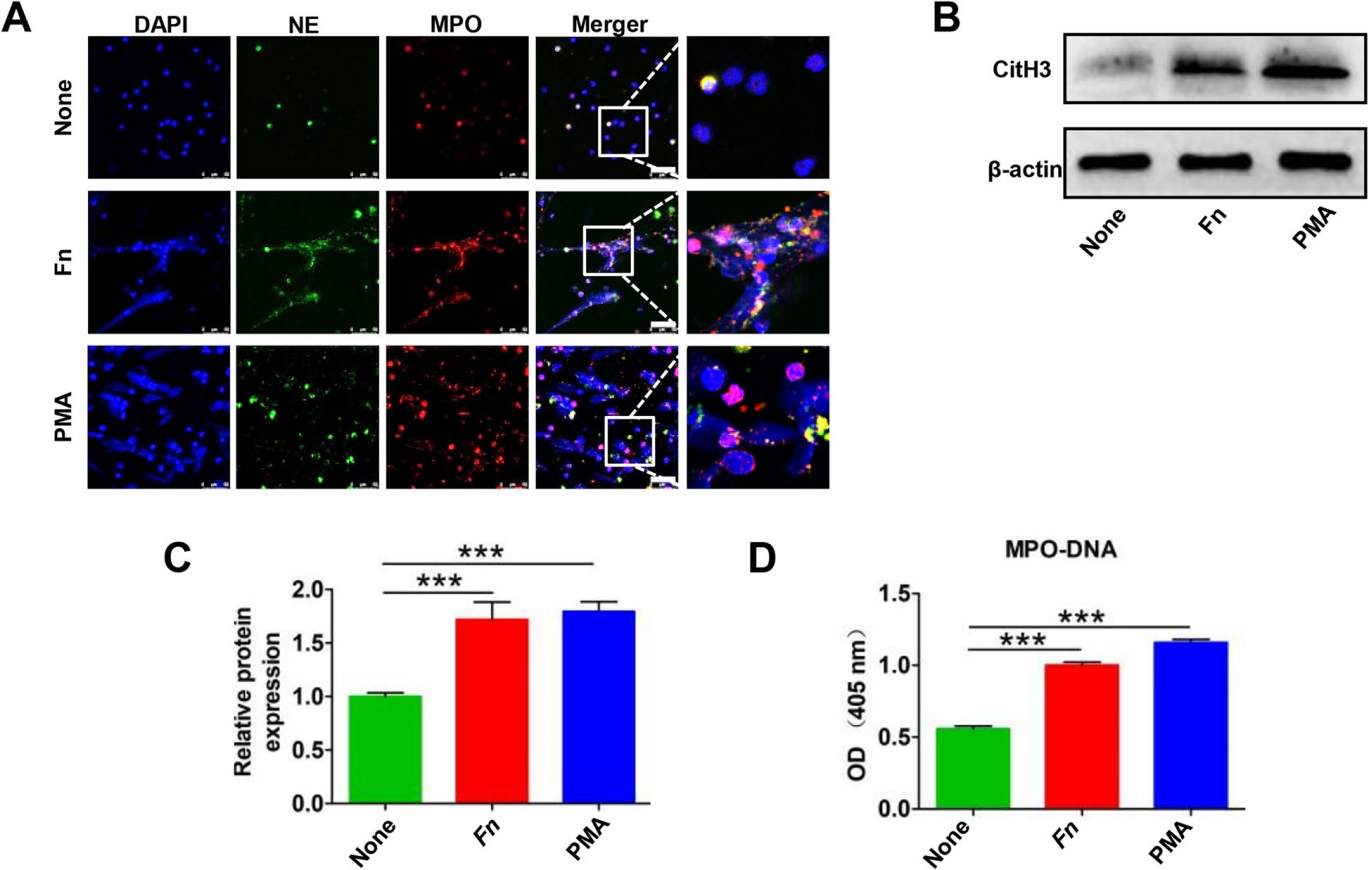
*Fn* facilitates NETs generation in vitro*.* **A** Representative IF images for DNA/NE/MPO in neutrophils treated with *Fn* or PMA for 4 h. **B** Western blot analysis of CitH3 expression in neutrophils treated with *Fn* or PMA for 4 h. **C** CitH3 protein levels were quantified by using densitometry and normalized to β-actin and are shown as fold changes compared to the control. Each bar displays the means ± SD of three independent experiments. **D** ELISA analysis of MPO-DNA levels in the supernatant of neutrophils treated with *Fn* or PMA for 4 h. None, no treatment. White scale bars: 50 μm. ***p < 0.001

Given the correlation between serum NETs levels and *Fn* DNA levels, we wondered whether *Fn* can stimulate neutrophils to produce and release NETs in vitro. NETs-associated markers were detected by IF staining (DNA/MPO/NE levels), western blotting (CitH3 expression) and ELISA (MPO-DNA levels). It had been widely reported that neutrophils can form and release abundant NETs upon PMA stimulation. Here, we used PMA stimulation as a positive control. Neutrophils stimulated with *Fn* showed obvious web-like NETs formation, as identified through morphological staining of DNA/MPO/NE (Fig. 2A). Similar trends were further verified by analysis of CitH3 expression in neutrophils and MPO-DNA levels in the supernatant of neutrophils stimulated with *Fn* (Fig. 2B-D).

### Effect of *Fn*-induced NETs on the survival of CRC cells in vitro

**Fig. 3:**
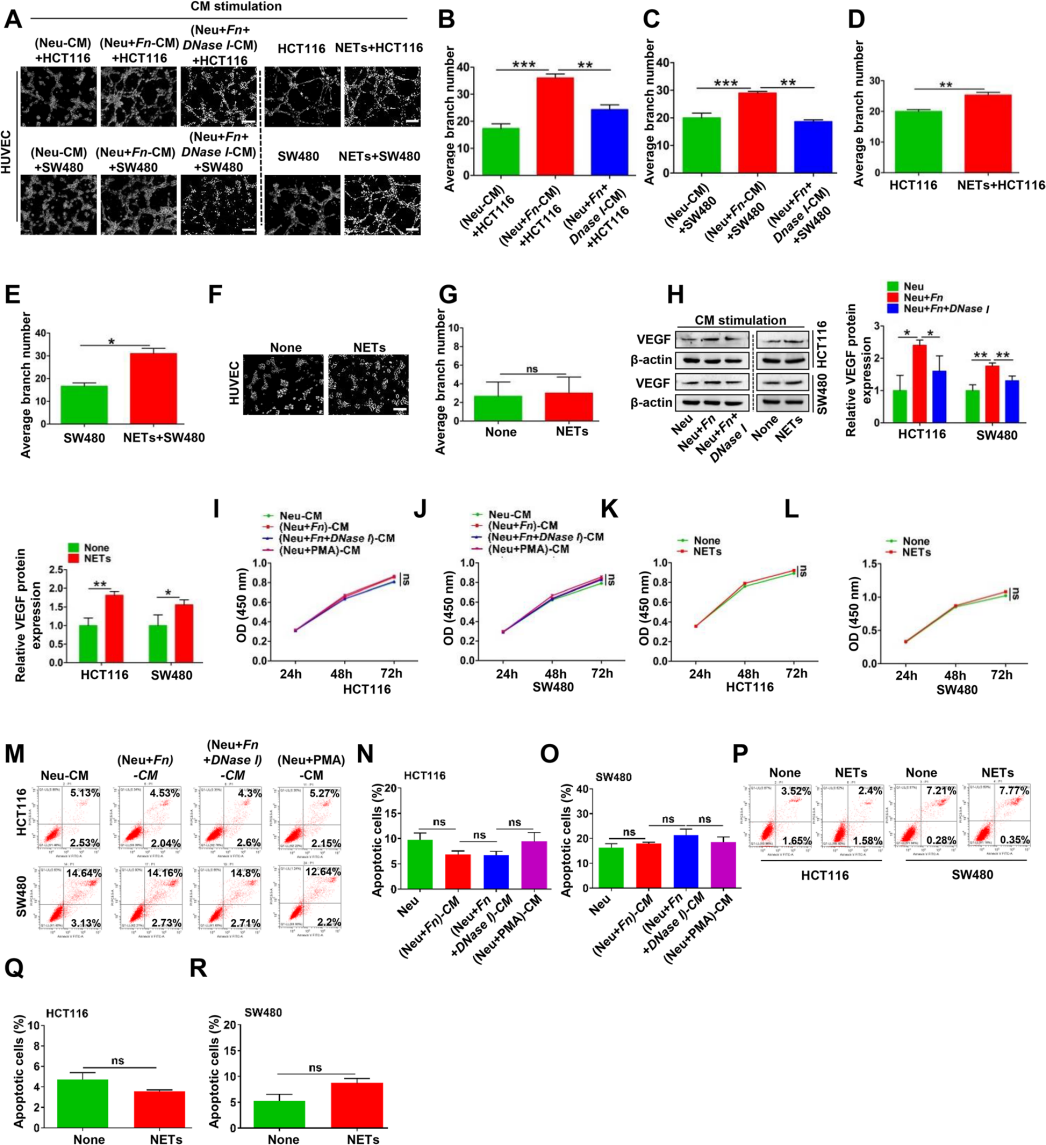
The effect of NETs on survival of CRC cells in vitro. **A**-**E** HUVEC tube formation assay using various CM from CRC cells treated with (Neu)-CM, (Neu + *Fn*)-CM, (Neu + *Fn* + *Dnase I*)-CM or with and without NETs for 4 h (**A**). Quantification analysis of the average branch number (**B**-**E**). **F**-**G** HUVEC tube formation assay treated with and without NETs for 4 h (**F**). Quantification analysis of the average branch number (**G**). **H** Western blot analysis of VEGF in CRC cells exposed to (Neu)-CM, (Neu + *Fn*)-CM, (Neu + *Fn* + *DNase I*)-CM or with and without NETs for 48 h. Protein levels of VEGF were quantified by using densitometry and normalized to β-actin and are shown as fold changes compared to the control (Right). Each bar displays the means ± SD of three independent experiments. **I**-**J** CCK8 assay for proliferation ability of CRC cells cultured with (Neu)-CM, (Neu + *Fn*)-CM, (Neu + *Fn* + *DNase I*)-CM, (Neu + PMA)-CM for 24, 48, and 72 h. **K**-**L** CCK8 assay for proliferation ability of CRC cells treated with or without NETs for 24, 48, and 72 h. **M**–**O** Apoptosis analysis for CRC cells treated with (Neu)-CM, (Neu + *Fn*)-CM, (Neu + *Fn* + *DNase I*)-CM for 48 h. Data are shown as means ± SD in three independent experiments (**N**–**O**). **P**-**R** Apoptosis analysis for CRC cells treated with or without NETs for 48 h. Data are shown as means ± SD in three independent experiments (**Q**-**R**). ns, not significant, White scale bars: 200 μm.*p < 0.05, **p < 0.01, ***p < 0.001

To examine whether *Fn*-induced NETs influence CRC cell survival by regulating tumor angiogenesis, proliferation and cell apoptosis, we used CM from *Fn*-treated neutrophils to stimulate HCT116 and SW480 cells and investigated their malignant behavioral changes as well as their effect on angiopoiesis. The tube formation of HUVECs was significantly increased in the group treated with CM either of the two CRC cell lines incubated with (Neu + *Fn*)-CM, while the increasing trend was abolished when *DNase I* was added to diminish NETs formation (Fig. 3A-C). Here, the intrinsic impact of *DNase I* on tube formation was also excluded (Fig. S1A). Likewise, the same results were obtained when HUVECs were treated with CM from either of the two CRC cell lines incubated with purified NETs (Fig. 3A and D-E). Surprisingly, there were few effects of direct NETs stimulation on HUVECs (Fig. 3F-G). In addition, we evaluated whether (Neu + *Fn*)-CM or purified NETs could regulate the expression of VEGF, an important angiogenic stimulator mainly derived from CRC cells. As expected, both treatments increased VEGF expression in the two CRC cell lines (Fig. 3H). Subsequently, we analyzed the effect of NETs on CRC cell proliferation, and found no obvious difference in the proliferative capability of CRC cells treated with either (Neu + *Fn*)-CM or purified NETs (Fig. 3I-L). Regarding apoptosis, we also found by Annexin V/PI double staining that the abovementioned treatments had no obvious influence on either of the two CRC cell lines (Fig. 3M-R).

### *Fn*-induced NETs facilitate metastasis of CRC in vitro

**Fig. 4:**
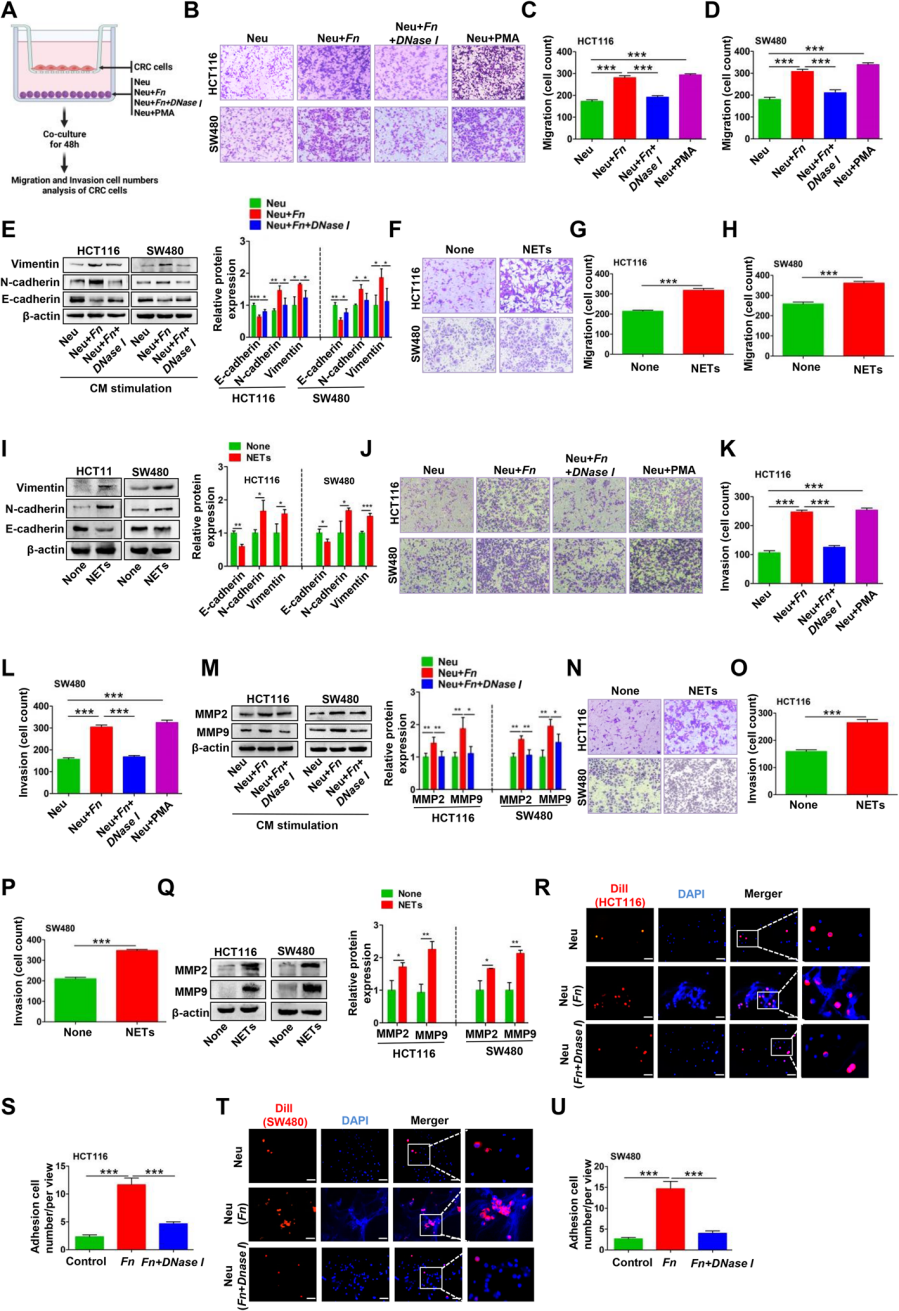
*Fn*-induced NETs facilitate metastasis of CRC in vitro. **A** The procedure of transwell migration and invasion assay for CRC cells co-cultured with (Neu), (Neu + *Fn*), (Neu + *Fn* + *DNase I*) and (Neu + PMA). **B**-**D** Transwell migration assay for CRC cells co-cultured with (Neu), (Neu + *Fn*), (Neu + *Fn* + *DNase I*) and (Neu + PMA) (B). Quantification analysis of the number of transmembrane cells (**C**, **D**). **E** Western blot analysis of E-cadherin, N-cadherin and vimentin in CRC cells treated with (Neu)-CM, (Neu + *Fn*)-CM, (Neu + *Fn* + *DNase I*)-CM for 48 h. Protein levels were quantified by using densitometry and normalized to β-actin and are shown as fold changes compared to the control (Right). Each bar displays the means ± SD of three independent experiments. **F**–**H** Transwell migration assay for CRC cells treated with or without NETs (F). Quantification analysis of the number of transmembrane cells (**G**-**H**). **I** Western blot analysis of E-cadherin, N-cadherin and vimentin in CRC cells treated with or without NETs. Protein levels were quantified by using densitometry and normalized to β-actin and are shown as fold changes compared to the control (Right). Each bar displays the means ± SD of three independent experiments. **J**-**L** Transwell invasion assay for CRC cells co-cultured with (Neu), (Neu + *Fn*), (Neu + *Fn* + *DNase I*), (Neu + PMA) (J). Quantification analysis of the number of transmembrane cells (K-L). **M** Western blot analysis of MMP9 and MMP2 in CRC cells treated with (Neu)-CM, (Neu + *Fn*)-CM, (Neu + *Fn* + *DNase I*)-CM for 48 h. Protein levels were quantified by using densitometry and normalized to β-actin and are shown as fold changes compared to the control (Right). Each bar displays the means ± SD of three independent experiments. **N**-**P** Transwell invasion assay for CRC cells treated with or without NETs (N). Quantification analysis of the number of transmembrane cells (**O**-**P**). **Q** Western blot analysis of MMP9 and MMP2 in CRC cells treated with or without NETs. Protein levels were quantified by using densitometry and normalized to β-actin and are shown as fold changes compared to the control (Right). Each bar displays the means ± SD of three independent experiments. **R**-**U** Adhesion assay for Dil-labeled CRC cells trapped within *Fn*-induced NETs in vitro. Representative fluorescence images were shown. White scale bars: 50 µm. The number of Dil-labeled CRC cells is quantified and shown in the right panel, respectively. White scale bars: 50 μm. *p < 0.05, **p < 0.01, ***p < 0.001

Migration, invasion and adhesive trapping assays were conducted to investigate the effects of *Fn*-induced NETs on the metastasis-related behaviors of CRC cells. Similar to the Neu + PMA coculture system, the Neu + *Fn* coculture system efficiently enhanced the migratory ability of HCT116 and SW480 cells, which was partially diminished after prevention of NETs formation by coculture with Neu + *Fn* + *DNase I* (Fig. 4A-D). Enhanced migration of epithelial cells is usually linked to EMT, and we then focused on the expression of EMT-related markers under the same conditions. Interestingly, incubation with (Neu + *Fn*)-CM decreased the epithelial marker E-cadherin and increased the expression of mesenchymal markers N-cadherin and vimentin, while these effects were reversed by coculture with (Neu + *Fn* + *DNase I*)-CM (Fig. 4E). Furthermore, purified NETs were verified to increase the migration and induce EMT in CRC cells (Fig. 4F-I). A similar trend was also verified by IF analysis of E-cadherin and Vimentin (Fig. S1I-J). Moreover, enhanced invasiveness of HCT116 and SW480 cells was observed using the abovementioned treatment system, which may be associated with increases in the levels of the invasion-associated proteins MMP2 and MMP9 induced by (Neu + *Fn*)-CM and purified NETs (Fig. 4J-M and N-Q). Here, the effects of PMA or *DNase I* alone on the migration and invasion abilities of CRC cells were excluded (Fig. S1C-H). We further showed the adhesive trapping capacity of NETs on CRC cells. In the adhesive trapping assay, we observed extensive NETs release from neutrophils stimulated with *Fn* and a dramatically increased adhesion rate of the two CRC cell lines. When *DNase I* was used to destroy the web-like structure of NETs, the number of adhered CRC cells decreased (Fig. 4R-U).

### *Fn*-induced NETs facilitate malignant growth and metastasis in vivo

**Fig. 5:**
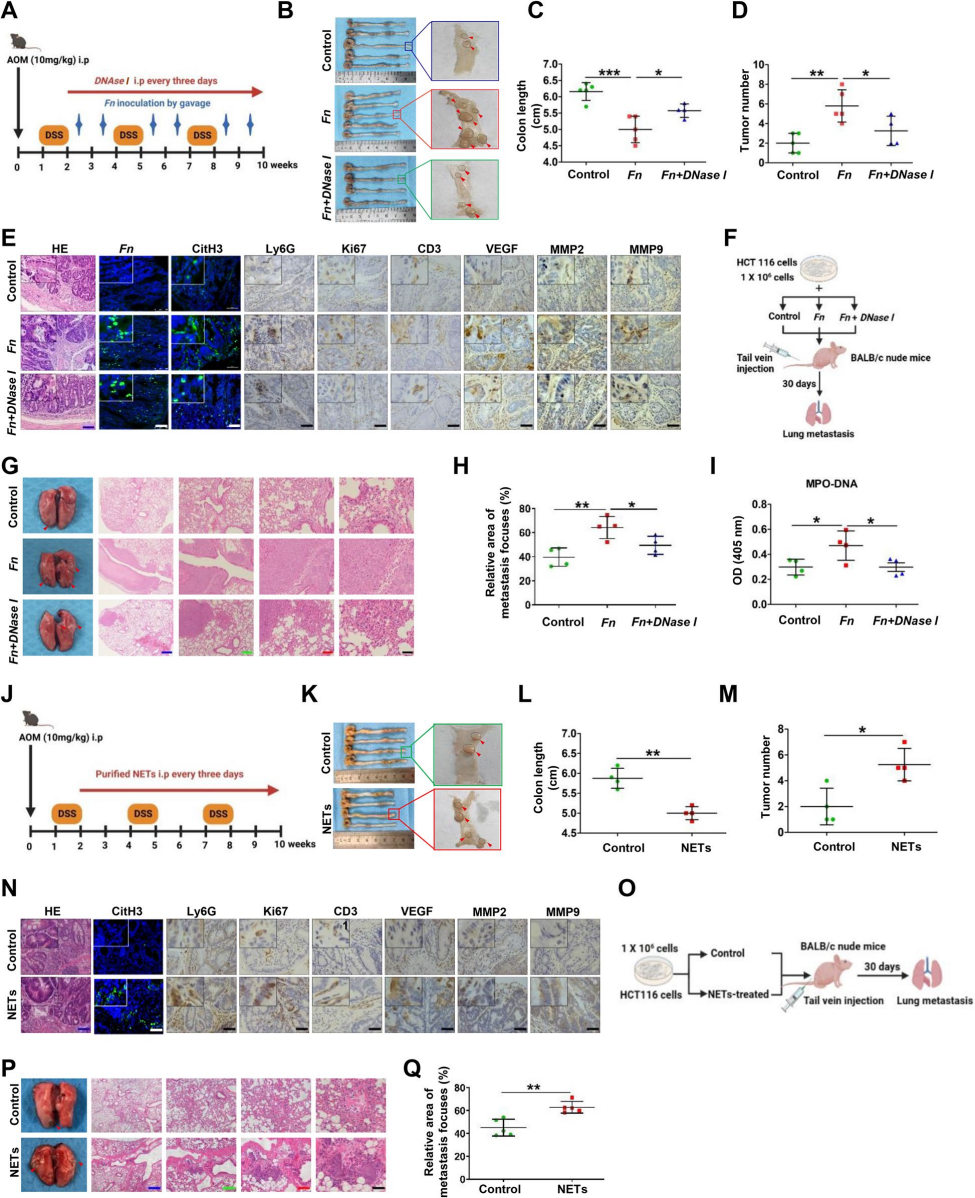
*Fn*-induced NETs facilitate malignant growth and metastasis in vivo*.* **A** The schematic diagram of *Fn* inoculation by gavage with and without *DNase I* treatment based on AOM/DSS mice model. **B** Representative images of the whole colon tissues (left panel) and the tumor nodules in colon tissues as indicated by the red arrows (right panel). **C** Colon length from all groups of mice was measured on week 12. Data are shown as means ± SD. **D** The colonic tumor nodules from all groups of mice were counted on week 12. Data are shown as the average tumor number ± SD. **E** Representative images of H&E staining, FISH for *Fn*, IF staining for CitH3 and IHC staining for Ly6G, Ki67, CD31, VEGF, MMP2 and MMP9 in representative colon tumor sections from AOM/DSS mice treated with or without *Fn* and *DNase I*. Blue scale bars: 100 µm. White scale bars and black scale bars: 50 µm. **F** The schematic diagram of CRC metastasis mice injected with HCT116 cells, HCT116 cells + *Fn* or HCT116 cells + *Fn* + *DNase I*. **G** Representative images of morphological features of macroscopic lungs of nude mice and the H&E staining of lung metastasis. Black scale bars, 50 µm; Red scale bars, 100 µm; Green scale bars, 200 µm; Blue scale bars, 400 µm. **H** Statistical analysis of metastasis focuses in lungs from individual mouse. **I** ELISA analysis for the serum levels of MPO-DNA in mice. **J** The schematic diagram of purified NETs treatment based on AOM/DSS mice model. **K** Representative images of the whole colon tissues (left panel) and the tumor nodules in colon tissues as indicated by the red arrows (right panel). **L** Colon length from all groups of mice (*n* = 4 / each group) was measured on week 12. Data are shown as means ± SD. **M** The colonic tumor nodules from all groups of mice (*n* = 4 / each group) were counted on week 12. Data are shown as the average tumor number ± SD. **N** Representative images of H&E staining, IF staining for CitH3 and IHC staining for Ly6G, Ki67, CD31, VEGF, MMP2 and MMP9 in representative colon tumor sections from AOM/DSS mice treated with or without purified NETs. Blue scale bars: 100 µm. White scale bars and black scale bars: 50 µm. **O** The schematic diagram of CRC metastasis mice injected with HCT116 cells or NETs-treated HCT116 cells. **P** Representative images of morphological features of macroscopic lungs of nude mice and the H&E staining of lung metastasis. Black scale bars, 50 µm; Red scale bars, 100 µm; Green scale bars, 200 µm; Blue scale bars, 400 µm. **Q** Statistical analysis of metastasis focuses in lungs from all groups of mice (*n* = 5 / each group). *p < 0.05, **p < 0.01, ***p < 0.001

We further explored whether *Fn* promotes tumor growth and metastasis by inducing NETs formation in vivo. Mice in the AOM/DSS-induced colitis-associated colorectal cancer (CAC) model were treated with *Fn* by oral gavage with or without NETs abrogation using *DNase I* (Fig. 5A). Shortening of the colon is a key indicator of the colitis severity. The colon length was observed to be significantly shorter in *Fn*-treated mice compared to control mice, and this effect was partially reversed by *DNase I* and its direct destruction of NETs (Fig. 5B and C). A significant increase in the tumor number was observed in the *Fn*-treated group, while *DNase I* had an inhibitory effect on the increase in the tumor numbers (Fig. 5B and D). Histological examination was performed to confirm the pathology of dysplasia in colorectal tissues. H&E staining revealed that the *Fn*-treated mice generally developed high-grade intraepithelial neoplasia (Fig. 5E). To determine whether NETs formation can be induced by *Fn* infection, we assessed *Fn* and CitH3 levels in the colonic sections by FISH and IHC, respectively. An abundantance *Fn* was validated in the AOM/DSS mice infected with *Fn* (Fig. 5E). Furthermore, consistent with the *Fn* abundance, elevated CitH3 was also oberved in the same colonic section (Fig. 5E). IHC analysis showed that the density of Ly6G^+^ neutrophil infiltration was higher in the tumors of AOM/DSS-treated mice injected with *Fn* than in those of the control group, indicating more neutrophil infiltration in *Fn-*infected CRC. The expression levels of the angiogenesis-promoting factors CD31 and VEGF, invasion-associated proteins MMP2 and MMP9 and proliferation marker Ki67 had similar trends (Fig. 5E and Fig. S2A).

To confirm whether *Fn* accelerates metastasis by NETs induction, *Fn* and HCT116 cells with or without *DNase I* were coinjected into nude mice via the tail vein for a lung metastasis assay (Fig. 5F). We observed increased numbers of metastatic nodules in the lungs after intravenous injection of *Fn* (Fig. 5G). In contrast, when *DNase I* was adopted to degrade the formed NETs, fewer metastatic nodules in the lung were observed (Fig. 5G-H). MPO-DNA levels in mouse serum were also measured by ELISA to demonstrate NETs formation. The NETs levels in the *Fn*-treated mouse group were clearly higher than those in the control group and the (*Fn* + *DNase I*)-treated mouse group (Fig. 5I).

In accordance with the abovementioned results, AOM/DSS-treated mice treated with purified NETs also exhibited higher tumor numbers as well as a shortened colon length (Fig. 5J-M). IF and IHC analyses showed similar trends (Fig. 5N and Fig. S2B). Moreover, the group of mice injected with purified NETs-treated HCT116 cells injection via the tail vein exhibited an increased metastatic burden in the lungs (Fig. 5O-Q).

### *Fn* stimulates NETs generation by activating TLR4-ROS signaling and the NOD1/2 receptor.

**Fig. 6:**
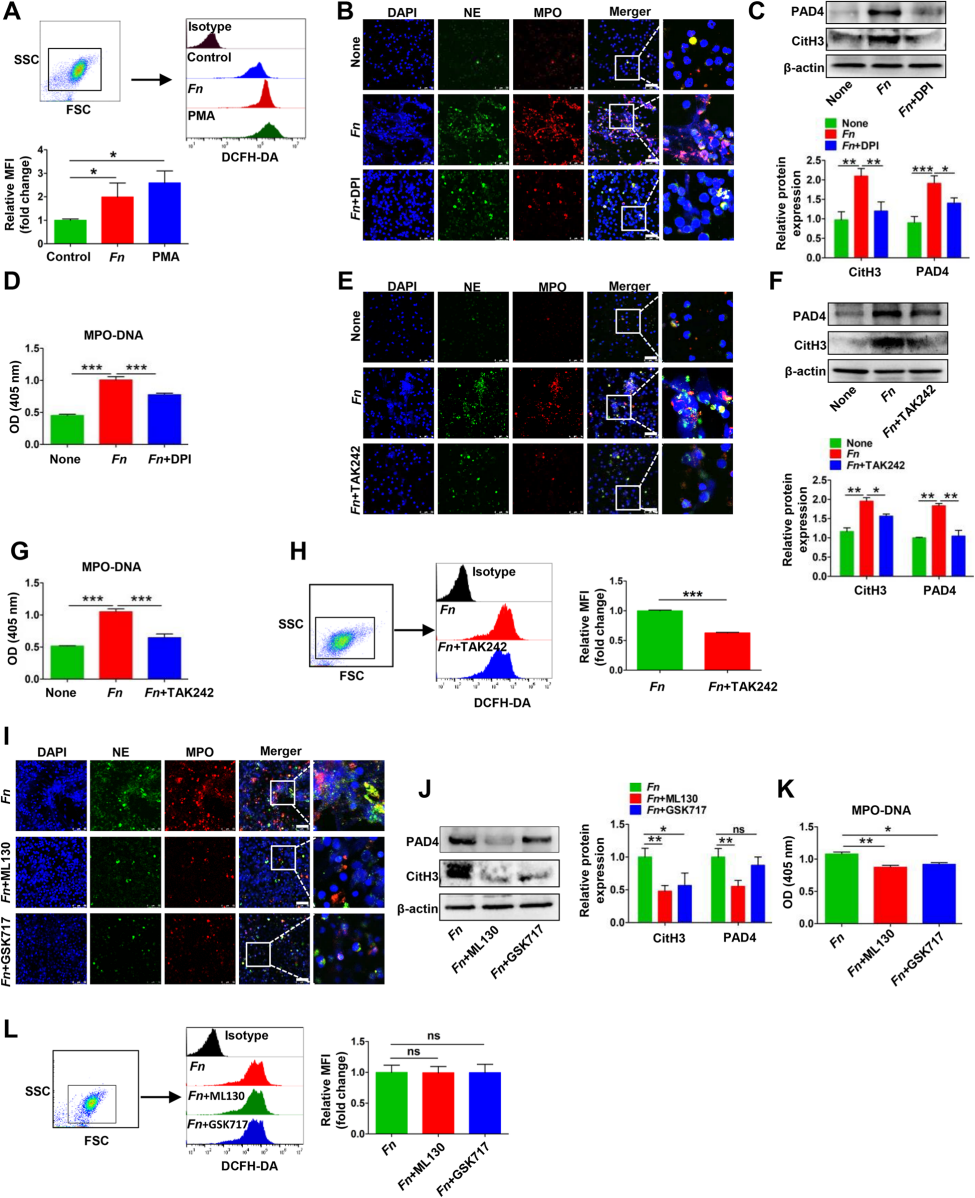
*Fn* stimulates NETs generation by activating TLR4-ROS signaling and NOD1/2 receptor. **A** Flow cytometry analysis for ROS levels in neutrophils treated with *Fn* or PMA for 8 h. The mean fluorescence intensity (MFI) of three independent experiments is quantified and shown below. **B** Representative IF images for DNA / NE / MPO in neutrophils treated with *Fn* or *Fn* + DPI for 8 h. **C** Western blot analysis of CitH3 and PAD4 in neutrophils treated with *Fn* or *Fn* + DPI for 8 h. Protein levels were quantified by using densitometry and normalized to β-actin and are shown as fold changes compared to the control (Right). Each bar displays the means ± SD of three independent experiments. **D** ELISA analysis was performed to detect MPO-DNA levels in the supernatant of neutrophils treated with *Fn* or *Fn* + DPI for 8 h. **E** Representative IF images for DNA / NE / MPO in neutrophils treated with *Fn* or *Fn* + TAK-242 for 8 h. **F** Western blot analysis of CitH3 and PAD4 in neutrophils treated with *Fn* or *Fn* + TAK-242 for 8 h. Protein levels were quantified by using densitometry and normalized to β-actin and are shown as fold changes compared to the control (Right). Each bar displays the means ± SD of three independent experiments. **G** ELISA analysis was performed to detect MPO-DNA levels in the supernatant of neutrophils treated with *Fn* or *Fn* + TAK-242 for 8 h. **H** Flow cytometry analysis was used to detect ROS levels in neutrophils treated with *Fn* or *Fn* + TAK-242 for 8 h. The MFI of three independent experiments is quantified and shown (Right). **I** Representative IF images for DNA / NE / MPO in neutrophils treated with *Fn*, *Fn* + ML130 or *Fn* + GSK717 for 8 h. **J** Western blot analysis of CitH3 and PAD4 in neutrophils treated with *Fn*, *Fn* + ML130 or *Fn* + GSK717 for 8 h. Protein levels were quantified by using densitometry and normalized to β-actin and are shown as fold changes compared to the control (Right). Each bar displays the average + SD of three independent experiments. **K** ELISA analysis was performed to detect MPO-DNA levels in the supernatant of neutrophils treated with *Fn*, *Fn* + ML130 or *Fn* + GSK717 for 8 h. **L** Flow cytometry analysis was used to detect ROS levels in neutrophils treated with *Fn*, *Fn* + ML130 or *Fn* + GSK717 for 8 h. The MFI of three independent experiments is quantified and shown (Right). None, no treatment. White scale bars: 50 μm. ns, not significant, *p < 0.05, **p < 0.01, ***p < 0.001

NETs formation is a stepwise process, in which ROS are reported to be indispensable modulators. Consistent with this report, the present data obtained by FCM analysis showed that the overall ROS levels in *Fn*-stimulated neutrophils were elevated (Fig. 6A). In contrast, treatment with the ROS scavenging agent DPI markedly reversed *Fn*-mediated NETs, as shown by NET marker analysis, indicating that ROS is partially responsible for *Fn*-mediated NETs formation (Fig. 6B-D). Because the formation of NETs can be triggered by microorganisms through TLR4, we then explored whether TLR4 participates in the formation of NETs driven by *Fn*. Neutrophils stimulated with *Fn* displayed elevated *TLR4* gene expression (Fig. S1K). Blocking TLR4 with TAK-242 abrogated *Fn*-induced NETs formation, as shown by NETs marker ananlysis (Fig. 6E-G). In addition, blocking TLR4 with TAK-242 obviously inhibited *Fn*-mediated ROS production, indicating that TLR4-ROS signaling partially mediates *Fn*-driven NETs formation (Fig. 6H). Since *Fn* was reported to interact with NOD-like receptors and then promote periodontitis process [23], we then focused on the NOD1/2 receptor and explored whether NOD1/2 can also participate in NETs formation driven by *Fn*. *NOD1/2* gene expression was upregulated in neutrophils stimulated with *Fn* (Fig. S1L-M). Both the NOD1 receptor inhibitor ML130 and the NOD2 receptor inhibitor GSK717 had suppressive effects on NETs formation triggered by *Fn*, indicating that activation of NOD1/2 signaling is also partially responsible for NETs formation driven by *Fn* (Fig. 6I-K). However, no obvious change in the ROS levels, which were increased by *Fn*, was observed when ML130 or GSK717 was added (Fig. 6L).

### The clinical significance of circulatory NETs in CRC patients

We explored the distribution of NETs in CRC patients with various clinicopathological characteristics (Table 2). No obvious differences were observed between patients of different sexes (male/female) or ages (< 60/ ≥ 60) or with different tumor locations (colon/rectum). However, patients with poorly differentiated tumors exhibited higher NET levels than those with well- and moderately differentiated tumors. Patients with advanced CRC (TNM III/IV) also showed higher NET levels than those with early-stage CRC (TNM I/II). In addition, patients with metastasis exhibited higher NET levels.

**Table 2:** Distribution of serum NETs in 95 CRC patients with various clinicopathological parameters

**Parameter**	Serum specimen (*n* = 95)		
	(n,%)	NETs (MPO-DNA)	*P* value
**Gender**			
Male (n,%)	52 (54.7%)	0.33 (0.19)	0.39
Female (n,%)	43 (45.3%)	0.27 (0.10)	
**Age**			
< 60 (n,%)	38 (40%)	0.33 (0.14)	0.41
≥ 60 (n,%)	57 (60%)	0.29 (0.09)	
**Location**			
Colon (n,%)	47 (49.5%)	0.32 (0.11)	0.27
Rectum (n,%)	48 (50.5%)	0.29 (0.12)	
**Differentiation**			
Poorly (n,%)	31 (32.6%)	0.34 (0.10)	0.001
Well + Moderately (n,%)	64 (67.4%)	0.26 (0.06)	
**TNM stage**			
I + II (n,%)	45 (47.4%)	0.25 (0.08)	0.001
III + IV (n,%)	50 (52.6%)	0.37 (0.12)	
**Metastasis**			
Present (n,%)	50 (52.6%)	0.39 (0.12)	0.001
Absent (n,%)	45 (47.4%)	0.26 (0.14)	

*Abbreviations*: *n* number of samples, *CRC* colorectal carcinoma, *CRP* colorectal polyps

**Fig. 7:**
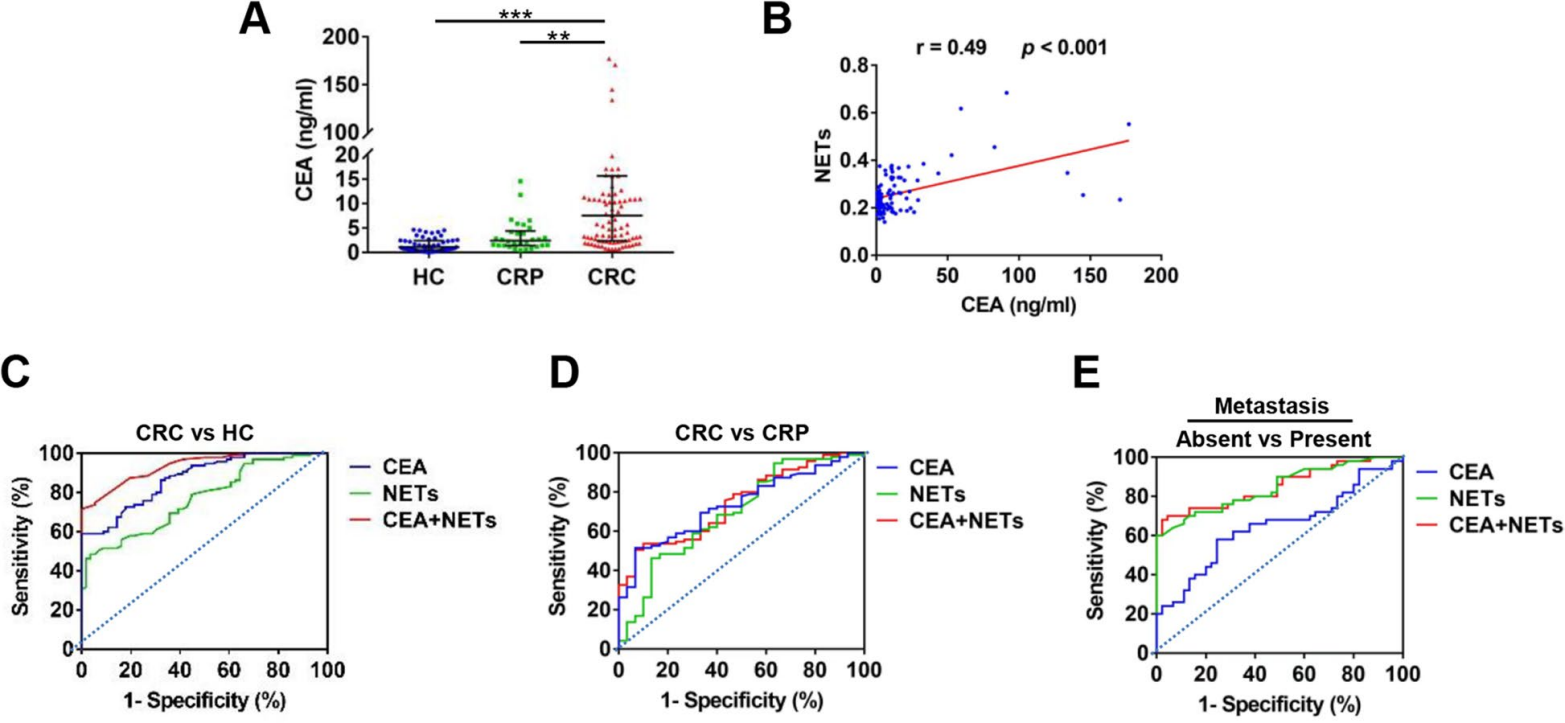
The clinical significance of CEA and circulatory NETs in CRC patients **A** The CEA levels in the serum from HCs (*n* = 56), CRP patients (*n* = 29) and CRC patients (*n* = 95). **B** Correlation between serum CEA levels and NETs levels in CRC patients. **C** ROC curve of serum CEA levels, NETs levels, and CEA combined with NETs levels for identifying CRC patients from HCs. **D** ROC curve of serum CEA levels, NETs levels, and CEA combined with NETs levels for identifying CRC patients from CRP patients. **E** ROC curve of serum CEA levels, NETs levels, and CEA combined with NETs levels for identifying metastasis in CRC patients. **p < 0.01, ***p < 0.001

CEA is widely used as a tumor marker in CRC for tumor detection and monitoring the response to therapy. We observed increased serum levels of CEA in CRC patients compared with those in CRP patients and HCs (Fig. 7A). Particularly, the level of circulating NETs was found to be positively correlated with the CEA level in CRC (Fig. 7B). Therefore, we evaluated the clinical significance of circulating NETs in CRC progression and metastasis. ROC analysis indicated that CEA, circulating NETs and CEA + NETs yielded AUCs of 0.87 (95% CI, 0.82–0.92), 0.746 (95% CI, 0.69–0.84) and 0.92 (95% CI, 0.89–0.97), respectively, for identifying CRC occurrence (Fig. 7C), suggesting that the combined assessment of CEA and NETs may be a potential screening strategy for CRC. ROC analysis indicated that CEA, circulating NETs and CEA + NETs had no better diagnostic efficacy for identifying CRC from CRP, with AUCs of 0.74 (95% CI, 0.64 to 0.82), 0.69 (95% CI, 0.58 to 0.80) and 0.74 (95% CI, 0.64–0.86), respectively (Fig. 7D). Moreover, CEA, circulating NETs and CEA + NETs yielded AUCs of 0.65 (95% CI, 0.54–0.76), 0.84 (95% CI, 0.77–0.92) and 0.85 (95% CI, 0.77–0.93), respectively, for identifying CRC metastasis (Fig. 7E). These data imply that circulating NETs alone or combined with CEA may be used as a potential biomarker for predicting CRC metastasis. Binary logistic regression analysis was further conducted to determine various influencing factors for CRC metastasis (Table 3). As expected, circulating NETs had a significant influence on metastasis (odds ratio [OR] = 6.76; 95% CI, 2.65–17.23; *p* = 0.001) in CRC.

**Table 3:** Binary logistic regression analysis for significant factors associated with metastasis in CRC

Parameter	ß	OR	95%CI	*P* value
**Gender**				
Male^a^	0.069	1.07	0.35–3.22	0.90
Female				
**Age**				
< 60^a^	0.006	1.00	0.96–1.05	0.79
≥ 60				
**Location**				
Colon^a^	0.042	1.04	0.36–3.05	0.93
Rectum				
**CEA**	0.016	1.02	0.99–1.04	0.30
**NETs (MPO-DNA)**	1.911	6.76	2.65–17.23	0.001
***Fn* abundance(-**△**Ct)**	-0.008	0.00	0.88–1.11	0.99

*Abbreviations*: *P* value < 0.05 was considered statistically significant. a, reference group, *ß* regression coefficient. *CI* confidence interval, *OR* odds ratio

## Discussion

**Fig. 8:**
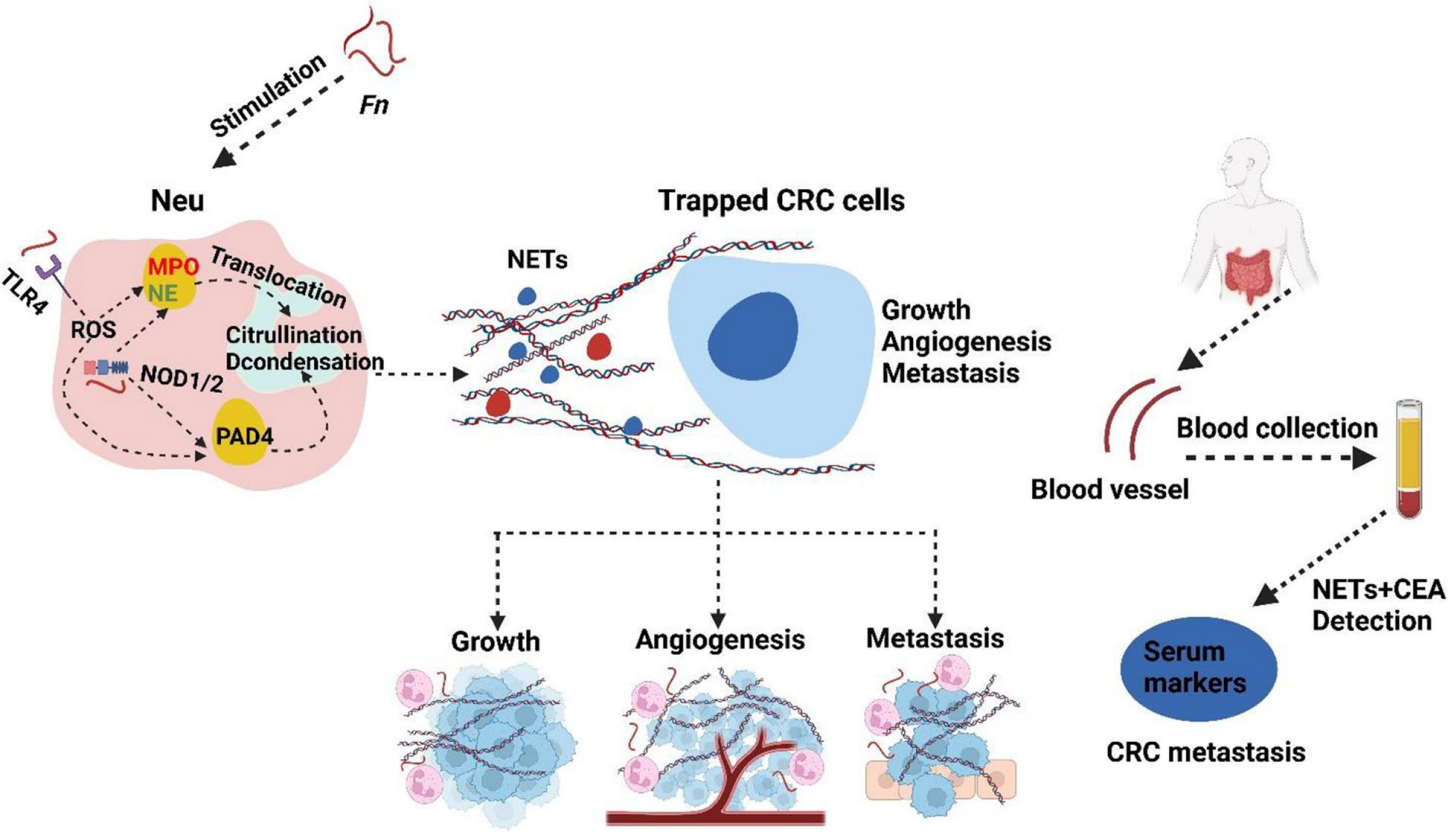
A working model illustrating that activation of TLR4-ROS and NOD1/2 signalings by *Fn*-mediated NETs formation, which subsequently facilitated the growth and metastasis of CRC cells. Additionally, monitoring circulating NETs, especially combined with CEA may have potential to be a biomarker for predicting CRC metastasis

Colorectal carcinoma refers to a group of heterogeneous tumors with a complex etiology. Dysbiosis of intestinal bacteria and the immune system have a profound influence on the neoplastic process. As one of the most vital enteropathogenic bacteria associated with CRC, *Fn* has enormous potential in activating immune cells and regulating the TME [24–26]. The detailed mechanisms underlying the interactions between *Fn* and the TME or immune system need to be further investigated. In the current study, we observed elevated NETs levels in CRC, especially in *Fn*^−high^ CRC. We clarified a regulatory role of *Fn* in neutrophil infiltration and NETs formation in CRC via activation of the TLR4-ROS and NOD1/2-dependent signaling pathways, thereby potentiating the growth and metastasis of CRC. In addition, the combined assessment of CEA and NETs may be a potential screening strategy for neoplastic progression of CRC (Fig. 8).

A number of studies have reported that tumor-infiltrating neutrophils in the TME participate in regulating the progression of CRC, while the mechanism by which tumor-infiltrating neutrophils regulate CRC remains poorly characterized [27]. Here, we observed a high intratumor density of CD66b^+^ neutrophils in CRC patients, especially in those with *Fn*^−high^ CRC, and this feature was also verified in colon tissue of *Fn*-gavaged mice. *Fn* was previously reported to selectively recruit myeloid-derived immune cells such as MDSCs, tumor-associated macrophages (TAMs), and dendritic cells (DCs) [28, 29]. Additionally, *Fn* creates a proinflammatory tumor milieu, which could induce CRC cells to upregulate the expression of IL-8 and CXCL1 and then recruit myeloid cells (mostly neutrophils) to the tumor [29]. Consistent with the literature, our present findings further suggested that the *Fn-*infected TME may be a crucial inducer of tumor-infiltration neutrophils in CRC.

Although the main function of NETs was initially found to be trapping and killing pathogens, NETs have garnered much attention in various noninfectious diseases especially in cancer [30]. Recent evidence has indicated that NETs are contributors to tumor initiation and progression [31]. Given the key indicator of *Fn-*induced infectious inflammation in CRC, we wondered whether *Fn* infection modulates the NETs formation, which subsequently facilitates CRC progression. Here, we provided compelling evidence that CRC patients with high enrichment of *Fn* (*Fn*^−high^ CRC) exhibited a higher abundance of NETs than patients with *Fn*^−low^ CRC, suggesting that *Fn* may be an elicitor of NETs generation in CRC. As expected, *Fn* induced neutrophils to form and release extensive web-like NETs in vitro. Importantly, an increasing number of studies have recently revealed that NETs participate in various pathogen infection-related cancers, including HBV-associated HCC [32], postoperative abdominal infection-associated gastric cancer [33], and human papillomavirus-associated cervical cancer [34]. Therefore, we speculate that NETs may be a promising intervention target for pathogen-associated tumors, a possibility that urgently needs to be verified in other infection-associated cancers.

An inflammatory TME favors the NETs formation, while uncontrolled production or overproduction of NETs reciprocally exacerbates inflammation and further facilitates tumor development [35]. Our present findings highlighted that *Fn*-induced NETs enhanced CRC malignancy via various tumor biological behaviors, including tumor growth and metastasis. With regard to tumor growth, we only observed increased proliferation of tumor cells in studies of *Fn*-infected mouse, and this increased proliferation may be mediated by intratumoral NETs, implying that NETs confer a growth advantage on the tumor in a TME-dependent manner. The present finding that increased tumor angiogenesis and VEGF levels in CRC cells stimulated by NETs may constitute one line of evidence. These results are consistent with the reported functions of NETs in regulating angiopoiesis via VEGF [36–38]. Regarding CRC metastasis, we comfirmed by in vitro and in vivo experiments that NETs formation by *Fn* boosted the migratory and invasive capacities of CRC cells, which may be associated with EMT occurrence, MMP2- and MMP9-mediated basement membrane protein degradation and trapping of CRC cells. In addition, inhibition of NETs by *DNase I* significantly reduced CRC growth and metastasis, suggesting that NETs could be a target for cancer intervention. Previous studies have reported that NETs accentuate cancer growth through the activation of the NF-κB and MAP kinase signaling cascade [39–41] and potentiate metastatic capacity by multiple effects, including cancer cell mobility, circulating tumor cell adhesion and promoting metastatic growth within the metastatic niche [42]. Therefore, the detailed mechanisms by which NETs induced by *Fn* facilitate CRC progression still need future study.

TLRs are specialized surface proteins expressed on the membrances of immune cells, including neutrophils, that can recognize the pathogen-associated molecular patterns (PAMPs) of gut microbes, trigger innate immune responses and prime antigen-specific adaptive immunity [43–45]. NETs formation mainly depends on the generation of ROS [46], which facilitates the release of MPO and NE from azurophilic granules and their transport to the nucleus, where NE cleaves histones and promotes chromatin decondensation [16, 47]. It has been reported that activation of the TLR4 pathway participates in ROS generation and cell damage [48]. Therefore, we focused on TLR4-ROS signaling to analyze the mechanism of *Fn*-induced NETs. Another type of chromatin decondensation is histone citrullination, which is driven by PAD4 [16, 49]. PAD4 lies downstream of ROS and calcium signaling during NETosis [50]. As expected, activation of TLR4-ROS signaling mediated *Fn*-induced NETs formation, which could influence the levels of PAD4 and MPO/NE. Moreover, NOD-like receptors play an important role in *Fn*-mediated periodontitis [23]. Therefore, in addition to focusing on plasma membrane surface receptors, we also focused on the role of the NOD1/2 receptor in NETs formation induced by *Fn*. In contrast to NOD1 activation, activation of NOD2 had no obvious effect on PAD4 expression, suggesting that NOD1-induced NETs formation was related to the activation of PAD4 and that NOD1 and NOD2 may both affect NETosis process via the release of MPO and NE. Studies have shown that inhibition of PAD2 or PAD4 significantly decreases sepsis-induced NETs formation [51]. Both PAD2 and PAD4 could be able to citrullinate histone H3 in the nucleus [52, 53]. Therefore, we suspect that NOD2-induced CitH3 may not be via PAD4 and whether PAD2 is involved in the process, which needs to be further studied.

CEA is a common biomarker in the diagnosis, metastasis monitoring and prognostic evaluation of CRC [54]. However, CEA has limited sensitivity and specificity for CRC and can be elevated in other malignancies [55]. Searching for novel noninvasive diagnostic biomarkers of CRC is thus of great importance. Our study revealed the involvement of NETs in CRC progression and a close correlation between serum NETs levels and CEA levels. In addition, accumulating evidence has shown that circulating NETs can entrap circulating tumor cells (CTCs) to facilitate cancer metastasis [56, 57], implying that NETs in peripheral blood may have predictive value as CTCs in predicting tumor progression. Given that circulating NETs can be readily detected, we analyzed their diagnostic value for the staging and metastasis of CRC, showing that circulating NETs, especially combined with CEA had the highest diagnostic value for predicting CRC occurence and metastasis. All this evidence suggests that the combination of CEA and circulating NETs could be a candidate marker for neoplastic progression of CRC.

In conclusion, the current findings suggested that elevated numbers of neutrophils and increased NETs levels were detected in CRC, especially in *Fn*^−high^ CRC. *Fn* induced neutrophils to form and release extensive NETs by activating TLR4-ROS signaling and the NOD1/2 receptor, which subsequently facilitated CRC growth and metastasis. Our findings also highlight monitoring circulating NETs, especially combined with CEA, as a novel biomarker strategy during neoplastic progression in CRC.

### Supplementary Information

**Additional file 1: Supplementary Figure S1.** (A-B) HUVEC tube formation assay treated with *DNase I *(A)*.* Quantification analysis of the average branch number (B). (C-E) Transwell migration assay for CRC cells treated with PMA or *DNase I *(C). Quantification analysis of the number of transmembrane cells (D-E). (F-H) Transwell migration assay for CRC cells treated with PMA or *DNase I *(F). Quantification analysis of the number of transmembrane cells (G-H). (I) Representative IF images for E-cadherin and Vimentin in CRC cells treated with (Neu)-CM, (Neu + *Fn*)-CM, (Neu + *Fn* + *DNase I*)-CM for 48 h. (J) Representative IF images for E-cadherin and Vimentin in CRC cells treated with or without NETs for 48 h. (K-M) qRT-PCR analysis for mRNA levels of *TLR4* (K), *NOD1* (L) and *NOD2* (M) in neutrophils treated with *Fn.* White scale bars: 50 μm. None, no treatment. ns, not significant, **p < 0.01, ***p < 0.001, **Supplementary Figure S2.** (A)The mean of IOD for Ly6G, Ki67, CD31, VEGF, MMP2 and MMP9 IHC staining were analyzed utilizing the Image Pro Plus software. Data were quantified by densitometry from 3 random fields of randomly selected 3 tissue sections from each group of mice and are shown as fold changes relative to the control. (B)The mean of IOD for Ly6G, Ki67, CD31, VEGF, MMP2 and MMP9 IHC staining were analyzed utilizing the Image Pro Plus software. Data were quantified by densitometry from 3 random fields of randomly selected 3 tissue sections from each group of mice and are shown as fold changes relative to the control. *p < 0.05, **p < 0.01

## Data Availability

The datasets used and analyzed during the current study are available within the manuscript and its additional files.

## Abbreviations

CRC: Colorectal cancer
HCs: Healthy controls
CRP: Colorectal polyps
CM: Conditioned medium
*Fn*: *Fusobacterium nucleatum*
MPO: Myeloperoxidase
NE: Neutrophil elastase
NETs: Neutrophil extracellular traps
PAD4: Peptidyl arginine deiminase 4
TLR: Toll-like receptor
NOD: NOD-like receptor
PMA: Phorbol 12-myristate 13-acetate
CitH3: Citrullinated modification of histone 3
EMT: Epithelial-mesenchymal transition
MMP: Matrix metalloproteinase
ROS: Reactive oxygen species
AUC: Area under the ROC curve
TME: Tumor microenvironment
Neu: Neutrophils
TAN: Tumor-associated neutrophil
VEGF: Vascular endothelial growth factor
HUVEC: Human umbilical vein endothelial cell
DPI: Diphenyleneiodonium
IF: Immunofluorescence
ELISA: Enzyme linked immunosorbent assay
IHC: Immunohistochemistry
PI: Propidium iodide
IOD: The integrated optical density
