# Supplementary material for: Fusobacterium nucleatum-triggered neutrophil extracellular traps facilitate colorectal carcinoma progression
Source: J Exp Clin Cancer Res. 2023 Sep 9;42:236. doi: 10.1186/s13046-023-02817-8 (PMC10492297; doi:10.1186/s13046-023-02817-8)
Supplement: Supplementary file 1 — Additional file 1: Supplementary Figure S1. (A-B) HUVEC tube formation assay treated with DNase I (A). Quantification analysis of the average branch number (B). (C-E) Transwell migration assay for CRC cells treated with PMA or DNase I (C). Quantification analysis of the number of transmembrane cells (D-E). (F-H) Transwell migration assay for CRC cells treated with PMA or DNase I (F). Quantification analysis of the number of transmembrane cells (G-H). (I) Representative IF images for E-cadherin and Vimentin in CRC cells treated with (Neu)-CM, (Neu + Fn)-CM, (Neu + Fn + DNase I)-CM for 48 h. (J) Representative IF images for E-cadherin and Vimentin in CRC cells treated with or without NETs for 48 h. (K-M) qRT-PCR analysis for mRNA levels of TLR4 (K), NOD1 (L) and NOD2 (M) in neutrophils treated with Fn. White scale bars: 50 μm. None, no treatment. ns, not significant, **p < 0.01, ***p < 0.001, Supplementary Figure S2. (A)The mean of IOD for Ly6G, Ki67, CD31, VEGF, MMP2 and MMP9 IHC staining were analyzed utilizing the Image Pro Plus software. Data were quantified by densitometry from 3 random fields of randomly selected 3 tissue sections from each group of mice and are shown as fold changes relative to the control. (B)The mean of IOD for Ly6G, Ki67, CD31, VEGF, MMP2 and MMP9 IHC staining were analyzed utilizing the Image Pro Plus software. Data were quantified by densitometry from 3 random fields of randomly selected 3 tissue sections from each group of mice and are shown as fold changes relative to the control. *p < 0.05, **p < 0.01 [file 13046_2023_2817_MOESM1_ESM.docx]

**Supplementary Materials**

***Fusobacterium nucleatum*-triggered neutrophil extracellular traps facilitate colorectal carcinoma progression**

Xuehua Kong^1,2^†, Yu Zhang^2^†, Linwei Xiang^1,2^†, Yan You^3^, Yaqian, duan^2^, Yuqing, Zhao^1^, Shue Li^4^, Rui Wu^5^, Jiangbo Zhang^6^, Lan Zhou^1^*, Liang Duan^2^*

1 Key Laboratory of Laboratory Medical Diagnostics, Ministry of Education, Department of Laboratory Medicine, Chongqing Medical University, Chongqing 400016, China

2 Department of Laboratory Medicine, The Second Affiliated Hospital of Chongqing Medical University, Chongqing 400010, China

3 Department of Pathology, The Second Affiliated Hospital of Chongqing Medical University,

Chongqing 400010, China

4 Department of Academic Research, The Second Affiliated Hospital of Chongqing Medical University, Chongqing 400010, China

5 Department of Laboratory Medicine, The First Affiliated Hospital of Chonqing Medical University, Chongqing 400016, China

6 Department of Gastrointestinal Surgery, The Second Affiliated Hospital of Chongqing Medical University, Chongqing, China

**†** These authors contributed equally to this work.

**^*^Corresponding authors**:

Liang Duan: duanliang@cqmu.edu.cn;

Lan Zhou: zhoulan@cqmu.edu.cn.


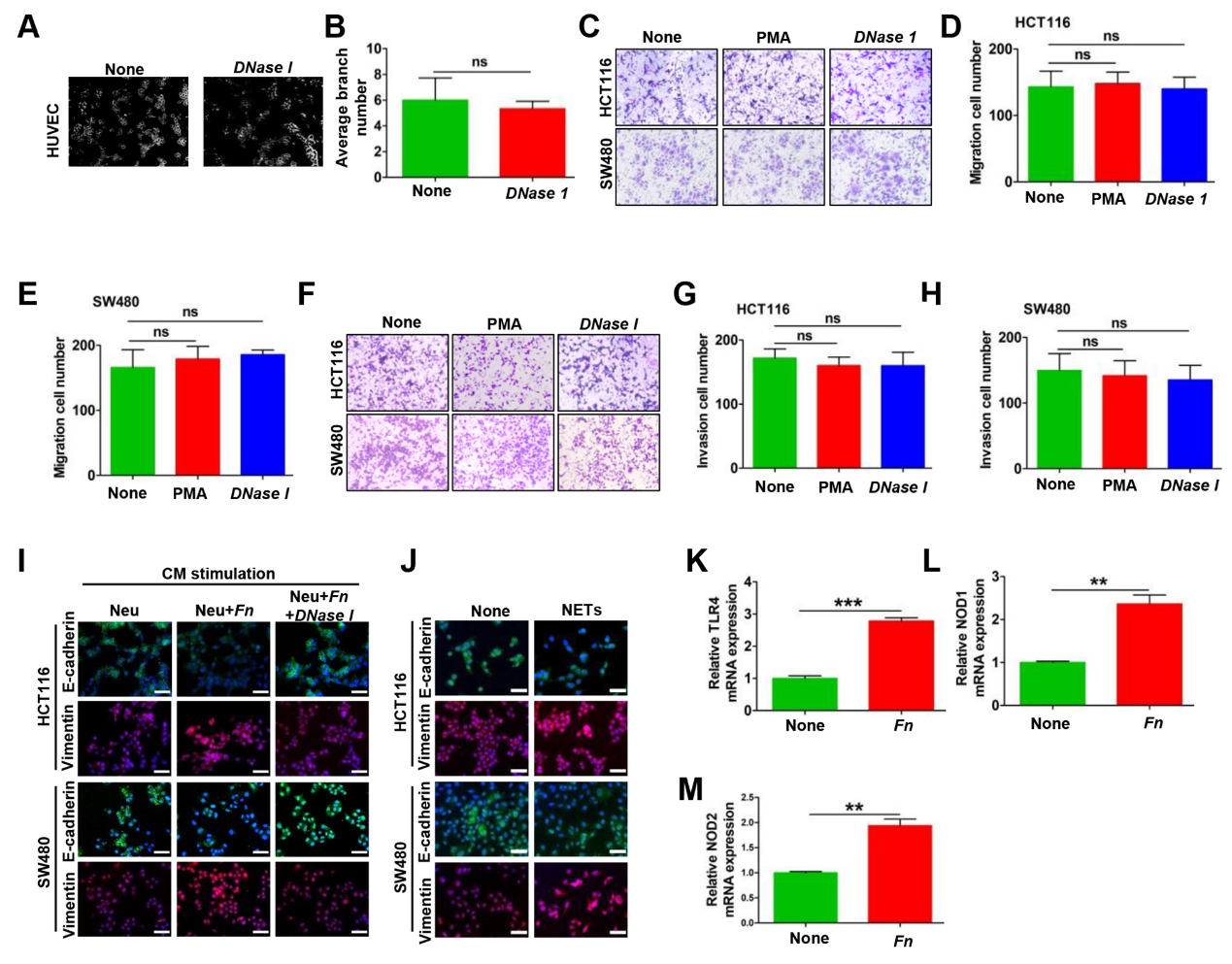


**Supplementary Figure S1**

(A-B) HUVEC tube formation assay treated with *DNase I* (A)*.* Quantification analysis of the average branch number (B).

(C-E) Transwell migration assay for CRC cells treated with PMA or *DNase I* (C). Quantification analysis of the number of transmembrane cells (D-E).

(F-H) Transwell migration assay for CRC cells treated with PMA or *DNase I* (F). Quantification analysis of the number of transmembrane cells (G-H).

(I) Representative IF images for E-cadherin and Vimentin in CRC cells treated with (Neu)-CM, (Neu + *Fn*)-CM, (Neu + *Fn* + *DNase I*)-CM for 48 h.

(J) Representative IF images for E-cadherin and Vimentin in CRC cells treated with or without NETs for 48 h.

(K-M) qRT-PCR analysis for mRNA levels of *TLR4* (K), *NOD1* (L) and *NOD2* (M) in neutrophils treated with *Fn.*

White scale bars: 50 μm. None, no treatment. ns, not signiﬁcant, ***p* < 0.01, ****p* < 0.001.


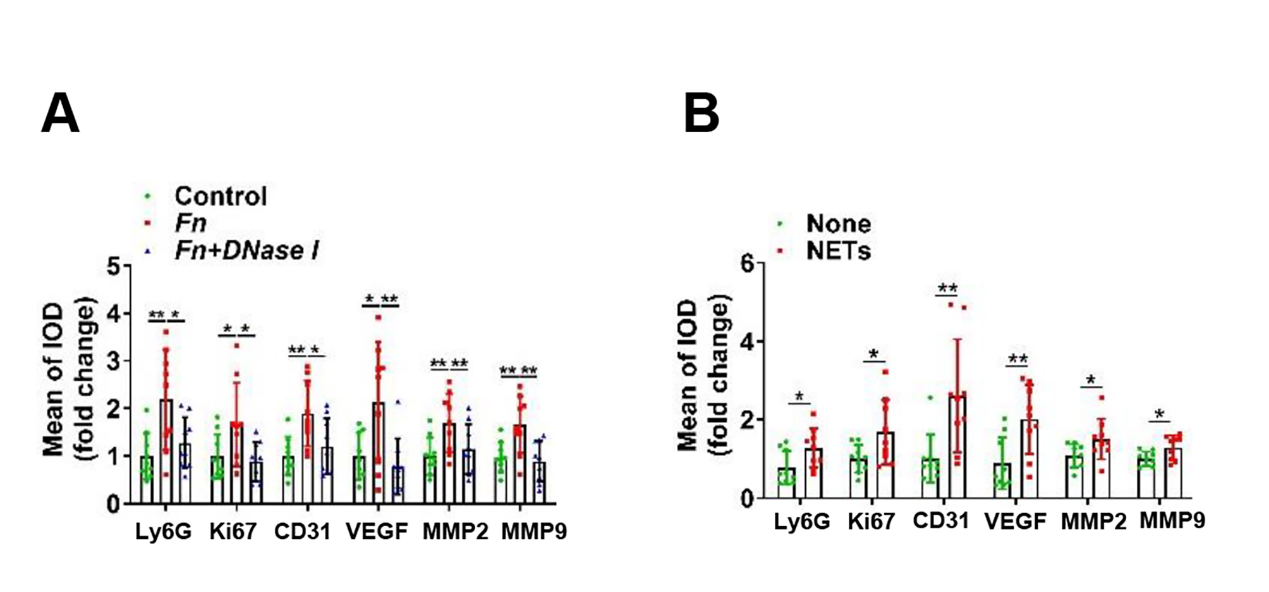


**Supplementary Figure S2**

(A)The mean of IOD for Ly6G, Ki67, CD31, VEGF, MMP2 and MMP9 IHC staining were analyzed utilizing the Image Pro Plus software. Data were quantified by densitometry from 3 random fields of randomly selected 3 tissue sections from each group of mice and are shown as fold changes relative to the control.

(B)The mean of IOD for Ly6G, Ki67, CD31, VEGF, MMP2 and MMP9 IHC staining were analyzed utilizing the Image Pro Plus software. Data were quantified by densitometry from 3 random fields of randomly selected 3 tissue sections from each group of mice and are shown as fold changes relative to the control.

**p* < 0.05, ***p* < 0.01.
